# How Well Do Popular Bicycle Helmets Protect from Different Types of Head Injury?

**DOI:** 10.1007/s10439-024-03589-8

**Published:** 2024-09-19

**Authors:** C. E. Baker, X. Yu, B. Lovell, R. Tan, S. Patel, M. Ghajari

**Affiliations:** 1 HEAD Lab, Dyson School of Design Engineering, Imperial College London, London, SW7 2AZ UK; 2 Department of Mechanical Engineering, University of Sheffield, Sheffield, S10 2TN UK

**Keywords:** Bicycle helmet testing, Brain injury risk, Brain injury prevention, Bicycle protective safety, Injury biomechanics, Bicycle helmet, Safety rating

## Abstract

Bicycle helmets are designed to protect against skull fractures and associated focal brain injuries, driven by helmet standards. Another type of head injury seen in injured cyclists is diffuse brain injuries, but little is known about the protection provided by bicycle helmets against these injuries. Here, we examine the performance of modern bicycle helmets in preventing diffuse injuries and skull fractures under impact conditions that represent a range of real-world incidents. We also investigate the effects of helmet technology, price, and mass on protection against these pathologies. 30 most popular helmets among UK cyclists were purchased within 9.99–135.00 GBP price range. Helmets were tested under oblique impacts onto a 45° anvil at 6.5 m/s impact speed and four locations, front, rear, side, and front-side. A new headform, which better represents the average human head’s mass, moments of inertia and coefficient of friction than any other available headforms, was used. We determined peak linear acceleration (PLA), peak rotational acceleration (PRA), peak rotational velocity (PRV), and BrIC. We also determined the risk of skull fractures based on PLA (linear risk), risk of diffuse brain injuries based on BrIC (rotational risk), and their mean (overall risk). Our results show large variation in head kinematics: PLA (80–213 g), PRV (8.5–29.9 rad/s), PRA (1.6–9.7 krad/s^2^), and BrIC (0.17–0.65). The overall risk varied considerably with a 2.25 ratio between the least and most protective helmet. This ratio was 1.76 for the linear and 4.21 for the rotational risk. Nine best performing helmets were equipped with the rotation management technology MIPS, but not all helmets equipped with MIPS were among the best performing helmets. Our comparison of three tested helmets which have MIPS and no-MIPS versions showed that MIPS reduced rotational kinematics, but not linear kinematics. We found no significant effect of helmet price on exposure-adjusted injury risks. We found that larger helmet mass was associated with higher linear risk. This study highlights the need for a holistic approach, including both rotational and linear head injury metrics and risks, in helmet design and testing. It also highlights the need for providing information about helmet safety to consumers to help them make an informed choice.

## Introduction

Cycling is an active mode of mobility with significant health and environmental benefits. In England, there has been a significant upward trend in cycling since 2002 [1]. Despite many health, environmental, and independent travel benefits, there can be a risk of trauma in bicycle falls and collisions. In Great Britain, cyclists had a reduction in fatalities in 2021 (down 21%) compared with a significant peak during the 2020 COVID-19 pandemic. Despite this reduction, cyclist fatalities in 2021 remained higher than the 2017 to 2019 average (increase of 17%) [2]. Head injuries are a key cause of fatal and life-changing injuries in cyclists [3]. Some cyclists choose to wear a helmet as a key line of defense against head injuries if they are involved in a collision or fall. Several previous studies, including a large meta-analysis of data relating to 64,000 cyclists, have shown that helmets have a protective effect to the head against head injury (including serious and fatal injury) and facial injury in cycle incidents, such as collisions and falls [3, 4]. In particular, helmet use has been found to reduce the risk of skull fractures, hemorrhages (extradural, subdural, subarachnoid, intraparenchymal, and intraventricular) and facial fractures when a cyclist is involved in a collision or fall event [5–13].

All helmets which come to market must pass a minimum safety threshold, set out by standards [14–20]. At the time of publication, current standards use metrics based on linear motion of the head to assess the safety of helmets. These metrics can assess the protection of helmets against head injuries caused by linear mechanisms, such as skull fractures and associated focal injuries, including extradural haematoma [21]. Rotational motion is also a known head injury mechanism, leading to diffuse brain injuries and hemorrhage [22–24]. New initiatives in the injury biomechanics field have led to a better understanding of rotational effects on brain injury outcomes, particularly diffuse brain injuries which are seen in cyclists [3]. Injured cyclists are commonly reported to lose consciousness [25, 26]. Loss of consciousness (LOC) is associated with rotational motion [23, 27]. LOC can occur across a broad range of head injury severities, with a range of long-term outcomes. In more severe instances, cyclists can sustain another rotationally driven injury, diffuse axonal injury (DAI), which often results in unfavorable long-term outcomes [28–30]. Cyclists also sustain subdural haematoma, which is strongly associated with rotational motion [11, 13, 22, 31]. Despite the importance of rotational effects and the broad range of head injuries sustained by cyclists, which can be attributed to them, rotational motion is not yet assessed in current standards.

A few recent studies have assessed the performance of bicycle helmets under oblique impacts, which better represent real-world incidents and produces larger head rotation [32–35]. These studies show a wide range of performance measured with metrics based on head translation and rotation, including peak linear acceleration (PLA), peak rotational acceleration (PRA), peak rotational velocity (PRV), and the brain injury criterion (BrIC) [36]. However, these studies use the HIII headform, which has biofidelity shortcomings. For example, the HIII headform has a large coefficient of friction, leading to overprediction of head rotational metrics [37]. A state-of-the-art, more biofidelic headform that can measure linear and rotational motion of the head more accurately has been developed recently. The key physical properties of this headform, including coefficient of friction, mass, and mass moments of inertia, better represent those of the medium human head than the HIII headform [37]. Hence, the new headform is better able to assess head protection performance of helmets. The development of the headform has been underpinned by international groups of experts at the CEN/TC158/WG11 working group to shape future helmet standards like cycle helmet standard, EN1078. This biofidelic headform provides a new opportunity for assessing the performance of bicycle helmets using more biofidelic lab test methods.

The aim of this study is to determine the performance of a range of bicycle helmets under oblique impacts using the new, biofidelic headform. We provide the ranges of linear and rotational head kinematics for 30 popular helmets used by cyclists in the UK. A widely adopted head rotation management technology is MIPS (Multi-direction Impact Protection System). Previous work with the Hybrid III headform has shown MIPS to provide protection against rotation [38, 39]. Here, we test whether this remains the case with the newly adopted headform, which has a lower coefficient of friction and more accurate moments of inertia than the HIII headform. In the absence of more objective ways to compare helmet performance, consumers may rely on helmet price as the indicator of safety [40, 41]. Hence, we also test whether there is a relationship between helmet price and its linear, rotational and overall performance. Mass is a factor considered to be important in the road cycling community. We therefore investigate whether mass influences linear, rotational or overall risk.

## Methods

**Fig. 1:**
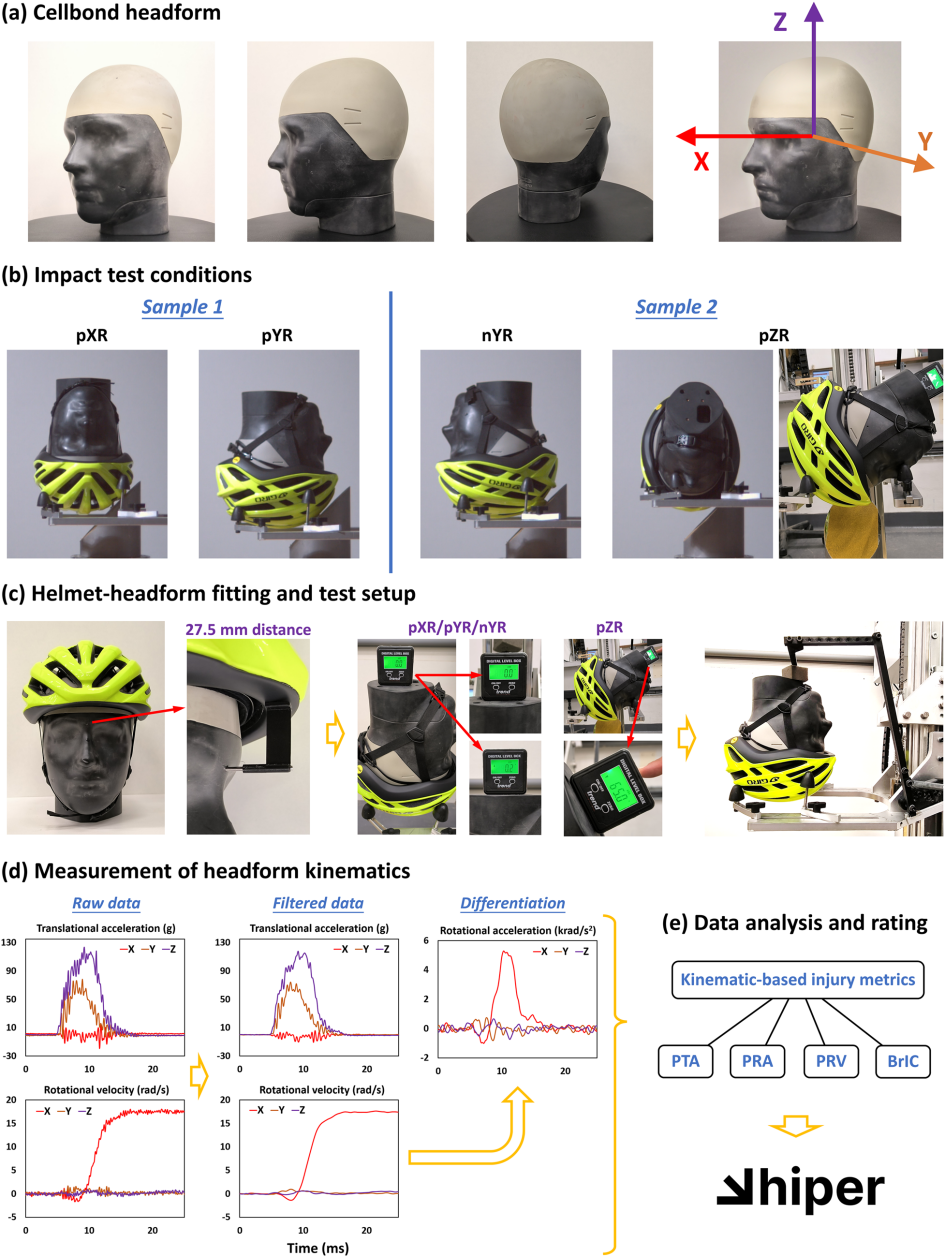
The test method summary. **a** The Cellbond-CEN 2022 headform. **b** Each helmet was tested at four locations. **c** For each test, three components of head linear acceleration and rotational velocity time-history were recorded. The rotational velocities were differentiated to obtain the rotational accelerations. **d** Data were analyzed to derive kinematics-based injury metrics, two of which were used to calculate the linear risk and rotational risk, with mean of these risks representing the overall risk

An overview of the test method is shown in Fig. 1. The following sections explain each component of the test method.

### The New Headform

For testing helmets, we used a new headform manufactured by Cellbond, a division of Encocam Limited, under the instruction of the European Committee for Standardization Working Group 11 (CEN/TC158/WG11). The Cellbond-CEN headform is made of nylon through an injection molding process. The significant advantage of this headform over previous headforms, such as the Hybrid III and EN960 headform, is its improved biofidelity in terms of the moments of inertia (MoIs) and coefficient of friction (CoF) of the headform surface (for more details please see [37]). MoIs and CoF of the headform are key factors in determining its rotational motion during helmeted head impacts [37, 42–44]. In a recent study, we compared the kinematics of an earlier version of the Cellbond-CEN headform with the Hybrid III headform in various oblique impact scenarios while wearing helmets [37]. The Cellbond-CEN headform produced much lower peak rotational kinematics than the Hybrid III headform. These results emphasize the importance of incorporating realistic MoIs and CoF to accurately assess helmet performance.

In this study, we used a version of the Cellbond-CEN headform, manufactured in 2022, which includes two further improvements (Fig. 1a): (i) the headform has a realistic head and face geometry derived from data of a large human population [37] and (ii) the headform has a small portion of the neck, in contrast to the HIII headform, ensuring a more realistic interaction between the chin strap and the neck. The physical properties of this version of the headform are provided in Table 1.

**Table 1:** The physical properties of the Cellbond-CEN 2022 headform

Circumference^1^ (mm)	Mass^2^ (kg)	MoI xx^3^ (kg cm^2^)	MoI yy^3^ (kg cm^2^)	MoI zz^3^ (kg cm^2^)	CoF^4^
570	4.25	196.7	225.9	144.4	0.40 ± 0.01

^1^The circumference is measured at a plane with a 10 degrees angle to the reference plane, which is 27.5 mm above the Frankfort plane

^2^The mass includes the instrumentation. The provided value is extracted from the CAD model of the headform

^3^MoI (Moments of Inertia) are measured at the center of gravity. The provided values are extracted from the CAD model of the headform

^4^CoF (Coefficient of Friction) is measured between the outer surface of the headform and a polyester strap. The measurement method is explained in [37, 45]. The measurements were made at the HEAD lab using the same machine

### Helmet Impact Conditions

The impact test conditions involved three factors: impact speed, impact angle, and impact location [46]. The values of these three factors were aligned with previous studies and supported by the findings in our recent, extensive literature review investigating cyclist head injury and impact characteristics [3, 32, 37, 45, 47]. Our literature review revealed that the head impact speed resulted from cyclist-ground impacts is concentrated around 6.5 m/s. In addition, the reviewed literature showed that the head impact angle (the angle between the impact velocity and the ground) is often between 30° and 60°. Hence, we chose a 6.5 m/s impact speed and a 45° impact angle.

Supported by previous studies and our literature review, we conducted helmet testing at four specific locations [3, 48–50]: left (pXR configuration), front (pYR configuration), rear (nYR configuration), and front-left (pZR configuration), as illustrated in Fig. 1b. Each impact location induced rotational motions predominantly about the specific axis of the headform which the impact location is named after.

### Helmet Impact Test Method

The oblique impact tests were conducted with the drop tower helmet test rig at the Human Experience, Analysis and Design (HEAD) Lab, Imperial College London. The test rig has been purposely built and previously used for testing helmets under various conditions [37, 46, 51]. The bicycle helmet (with visor attached if applicable) was securely fitted onto the headform following the manufacturer’s instructions. To ensure consistency, a distance of 27.5 mm was maintained between the edge of the helmet and the upper edge of the eye orbit marked on the headform (Fig. 1). The chin strap was then tightened in accordance with normal usage. Prior to tightening, a rigid cylinder with a diameter of 10 mm was placed between the chin and the strap and subsequently removed once the strap was tightened.

Next, the helmeted headform was positioned on a free-falling U-shape testing platform. To ensure proper alignment, we used an inclinometer to adjust the orientation of the headform (Fig. 1a). The goal across all test configurations was to achieve a nearly horizontal position with an inclination of 0° ± 1° for pXR/pYR/nYR or an inclination of 65° ± 1° for the pZR test. We then used a gripper to hold the helmet onto the platform, maintaining the helmet’s position and orientation during the free fall (Fig. 1a). The gripper was released by a mechanical trigger just prior to the helmet-anvil impact.

Helmets were dropped onto a metal anvil, with a 130 mm diameter and 45° inclined surface. The anvil was covered with an 80-grit abrasive paper to simulate the road surface, as suggested in previous studies and helmet test standards [46, 52–54]. The impact speed was recorded using a photoelectric sensor trigger system, which also triggered the video capture. A high-speed video camera was placed behind the anvil to record the impacts at a rate of 3500 frames per second. Following each test, the high-speed video was examined to verify that the helmet maintained its intended orientation and position on the platform until impact with the anvil.

For each helmet model, we conducted the four impact tests using two helmet samples. pXR and pYR were performed on helmet sample 1, and nYR and pZR were performed on helmet sample 2. The closest impact points (i.e., nYR and pZR) are at least 135° apart from each other. This is to ensure that the impact locations were separated from each other to minimize the influence of accumulated damage on the subsequent tests. To enhance the reliability of our results, each test was repeated three times using three different samples. Therefore, each helmet required 12 tests performed on 6 samples.

### Kinematic Data Capture and Processing

The headform was equipped with a DTS 6DX PRO sensor package along with a wireless datalogger system. This sensor package enabled the measurement of linear accelerations and rotational velocities along the three axes (Fig. 1c). Linear accelerations and rotational velocities were measured for 0.5 s either side of the time of impact at a sampling frequency of 20 kHz. All linear accelerations were filtered at CFC600 and rotational velocities were filtered at CFC180 according to ISO 6487 [55]. All signals were filtered before they were combined or used to derive other kinematic metrics, including calculating the resultant values and obtaining rotational acceleration via differentiating rotational velocity (Fig. 1d). We investigated N-point moving average to differentiate the filtered rotational velocity to obtain rotational acceleration. There was minimal difference between the peak rotational acceleration values obtained via the 1-point (no smoothing), 3-point, and 5-point moving averages, and therefore 1-point was adopted. Python was used to filter and differentiate the test data and perform subsequent analysis. The filtering was conducted using a fourth order Butterworth phaseless digital filter function (written according to SAE J211-1) [56]. The differentiation was done without any smoothing using the numpy library “gradient” function [57]. We extracted peak values of linear and rotational acceleration (PLA and PRA), rotational velocity (PRV), and BrIC. The mean value, averaged across all repeats for a given helmet and test configuration, were calculated as well as the standard deviation and coefficient of variation (CV).

### Injury Risk Calculation

Helmeted cyclists sustain both focal and diffuse brain injuries [3, 4]. Hence, we incorporated methods for evaluating the risk of these injuries from the measured head kinematics (Fig. 1d). We used the peak linear acceleration (PLA) to predict the risk of skull fractures and associated focal injuries. The focal injury risk function was based on a recent work where 30 elderly vulnerable road user collisions were reconstructed and a risk function for PLA at AIS4+ severity was produced [58]. Out of the 20 cases with AIS4+, 19 suffered skull fracture, SAH or contusions, which were predicted by PLA. Since the risk function was established using data from older casualties (60+), we adjusted it by multiplying PLA by the ratio of PLA of older population to the general population at 50% risk of AIS4+. We used the threshold adopted by helmet standards for the latter, i.e., 250 g [59] and 200 g for the former [60], leading to the following risk function:

1 $$P\left(\text{linear}\right)=\frac{1}{\left[1+{e}^{\left(3.3202-0.01312*\text{PLA}\right)}\right]}$$

where

2 $$\text{PLA}=\text{max}\left(\sqrt{{{a}_{x}\left(t\right)}^{2}+{{a}_{y}\left(t\right)}^{2}+{{a}_{z}\left(t\right)}^{2}}\right).$$

${a}_{x}(t)$, ${a}_{y}(t)$, and ${a}_{z}\left(t\right)$ represent the components of the head linear acceleration measured at the CoG.

We also used the injury metric based on head rotational velocities, BrIC, to predict the risk of diffuse injuries [36]. It has been shown that BrIC has a strong correlation with the maximum principal strain within the brain, produced in cycle helmet oblique impacts [61]. In addition, brain strain is shown to predict diffuse brain injuries, including white matter damage [62–65]. Risk functions have been developed for BrIC based on different definitions of brain strain (maximum principal strain, or MPS, and cumulative strain damage metric). These risk curves have been produced based on large animal experiments scaled to the human. The original risk curves were developed for severe diffuse brain injuries observed in animals, which were assumed to be of AIS4+ severity. These curves were scaled to obtain risk curves for other severities. We used the MPS-based BrIC risk function for AI2+ severity to evaluate the performance of helmets in preventing diffuse brain injuries [36]. This function is as follows:

3 $$P\left(\text{rotational}\right)=1-{e}^{-{\left(\frac{\text{BrIC}}{0.602}\right)}^{2.84}},$$

where

4 $$\text{BrIC}=\sqrt{{\left(\frac{\text{max}(|{\omega }_{x}\left(t\right)|)}{{\omega }_{xC}}\right)}^{2}+{\left(\frac{\text{max}(|{\omega }_{y}\left(t\right)|)}{{\omega }_{yC}}\right)}^{2}+{\left(\frac{\text{max}(|{\omega }_{z}\left(t\right)|)}{{\omega }_{zC}}\right)}^{2}.}$$

${\omega }_{x}\left(t\right)$, ${\omega }_{y}\left(t\right)$, and ${\omega }_{z}\left(t\right)$ are the components of the head rotational velocity. ${\omega }_{xC}$ = 66.25 rad/s, ${\omega }_{yC}$ = 56.45 rad/s, and ${\omega }_{zC}$ = 42.87 rad/s are the critical values of rotational velocity. These critical values were calculated such that they corresponded to 50% risk of severe diffuse axonal injuries in large animals [36].

After evaluating the risk of focal (linear) and diffuse (rotational) injuries for each impact location, we combined them to obtain an overall risk for each impact location. As there is currently insufficient data available to provide accurate weighting of these two types of injuries [3], we allocated them a 0.5 weighting to determine an overall injury risk for each impact location:

5 $$P\left(\text{injury }@ \text{location}\right)=0.5P\left(\text{linear }@\text{ location}\right)+0.5P\left(\text{rotational }@\text{ location}\right)$$

Finally, we used the weighting of each impact location determined from a meta-analysis of real-world cycle incident data to determine the overall injury risk for the helmet:

6 $$P\left(\text{injury }@\text{ helmet}\right)={\sum }P\left(\text{injury }@\text{ location}\right)\times P\left(\text{location}\right).$$

The meta-analysis included three studies that followed the same convention for defining the impact location, with a total number of 815 impact locations recorded [3]. The analysis provided the impact frequency for different helmet regions, namely front, side, rear, and crown. It showed that the crown is the least frequently impacted area. We used the weighting for the four impact configurations, pXR, pYR, nYR, and pZR. The pYR and pZR are both within the front region of the helmet as defined in the meta-data analysis. Hence, we equally split the weighting of front impacts between them. The weightings are provided in Table 2.

**Table 2:** The probability of impact location as determined from our previously published meta-analysis [3]

Location	pXR (side)	pYR or pZR (front)	nYR (rear)
$P\left(\text{location}\right)$	0.287	0.191	0.203

### Selecting Popular Helmets

Two approaches were combined to select a cohort of 30 helmets that included the most popular helmets purchased by UK consumers. Firstly, we used best-selling lists of major retailers, which yielded 10 helmets. 3 additional helmets with an additional protective technology, MIPS, were selected from the best-selling list to provide comparison. We additionally surveyed a broad cyclist population to yield a further 17 helmets. Importantly, all these helmets meet the European standard EN 1078.

### Helmets Selected from Popular Retailers’ Best-Selling Lists

Halfords and Decathlon are two of the largest in-store and online helmet providers in the UK. On each respective retailer’s website, adult cycling helmets were sorted using the “Best-Selling” function and the popularity of each helmet by website was recorded accordingly as a “Selling Rank” (as accessed in May 2022). Mountain biking-only, full-face, and time-trial helmets were removed from the longlist, resulting in a list of 76 helmets. The top 10 best-selling helmets were identified from the combined best-selling list (Table 3). In addition, 3 helmets from the top 10 had counterpart models which use MIPS, namely Lazer Compact DLX MIPS, Giro Angon MIPS, and Lazer Tonic MIPS. These helmets were additionally included to allow direct comparison between MIPS and non-MIPS versions of the same helmet.

**Table 3:** The top 10 best-selling cycling helmets were determined from a combined best-selling list from popular, major UK helmet retailors, Halfords, and Decathlon with three additional counterpart models which were fitted with MIPS also selected to give the 13 helmets listed

Brand	Model	Retailer
ABUS	Ace 2.0	Decathlon
BTwin	100 City	Decathlon
Btwin	500 City	Decathlon
Giro	Angon	Decathlon
Giro	Angon MIPS	Decathlon
Halfords	Essential	Halfords
Halfords	Sport	Halfords
Halfords	Trail	Halfords
Halfords	Urban	Halfords
Lazer	Compact	Halfords
Lazer	Compact MIPS	Halfords
Lazer	Tonic	Halfords
Lazer	Tonic MIPS	Halfords

### Cyclist Helmet Use Survey Design and Distribution for Helmet Selection

We surveyed UK cyclists to determine more information about their cycling habits, including helmet use and preferences. In line with Imperial College London’s ethics requirements, a survey was approved by the Head of Department for the Dyson School of Design Engineering and Research Governance Integrity Team (RGIT). The survey was circulated widely across various social media platforms to a large range (> 50) of online groups designed for cyclists. A significant effort was made to distribute the survey to groups attracting and consisting of a range of geographic locations, ages, genders, races, and other demographics. The survey invited any cyclists who owned or wore a helmet any proportion of the time to list the helmet make and model they use (if known). The following questions were used to obtain information about UK cyclist helmet-related preferences:

1. Do you wear a helmet when you cycle?
2. Do you own a cycling helmet?
3. What is the retail price of your current helmet?
4. What is the model name and brand of your current cycle helmet?

### Cyclist Helmet Survey Responses Informing Helmet Selection: Helmets and Their Prices

Of 1132 respondents, 1060 (93.6%) wore a helmet at least some of the time. Interestingly, a greater proportion owned a helmet (1083, 95.7%) all of whom provided information about the current retail price of the helmet. Around half (532/1083, 49.1%) of respondents who owned helmets provided information about the make or model of helmet. A frequency distribution was created, resulting in a list of popular helmets among survey respondents. Seven of the helmets already selected via the best-selling lists were present. Secondly, the survey results were used to better understand the price of helmets commonly worn by respondents. For each unique listed helmet, a corresponding retail price at the time of purchase selection (May 2022) was identified. The continuous distribution was subsequently classified into price bands, allowing for a distribution of cycling helmet prices to be obtained. This price distribution was then populated with the most popular road cycling helmets highlighted in the survey. Further details including figures can be found in the Appendix. The Btwin 100 City (£9.99) ranked eighth was unable to be purchased. This was replaced with another popular helmet, the Specialized Tactic MIPS (shown at the bottom of Table 4).

**Table 4:** List of 30 popular UK helmets selected for testing (based on manufacturer best-selling lists and survey responses) showing purchase price at the time of purchase (May 2022)

	Brand	Model	Price (£)	Mass (g)	Additional Technologies	Retailer
1	Halfords	Trail	£25.00	280	N/A	Halfords
2	Halfords	Sport	£15.00	240	N/A	Halfords
3	Giro	Angon	£64.99	280	N/A	Decathlon
4	Lazer	Compact	£30.00	325	N/A	Halfords
5	ABUS	Villite Ace 2.0 City	£89.99	400	Built-in LED	Decathlon
6	Halfords	Essential	£10.00	240	N/A	Halfords
7	Lazer	Tonic	£40.00	230	N/A	Halfords
8^a^	Btwin	100 City	£9.99	250	N/A	Decathlon
9	Halfords	Urban	£25.00	450	N/A	Halfords
10	Btwin	500 City	£24.99	560	N/A	Decathlon
11	Lazer	Compact DLX MIPS	£54.99	325	MIPS	Halfords
12	Giro	Angon MIPS	£79.99	280	MIPS	Decathlon
13	Lazer	Tonic MIPS	£65.00	230	MIPS	Halfords
14	Van Rysel	Road R500	£29.99	275	N/A	Decathlon
15	Bontrager	Solstice	£18.00	310	N/A	Sigma Sports
16	Specialized	Align MIPS	£31.00	355	MIPS	Sigma Sports
17	MET	Idolo	£25.00	255	N/A	Sigma Sports
18	MET	Crossover	£25.00	295	N/A	Wiggle
19	DHB	R2.0	£25.00	273	N/A	Wiggle
20	Mavic	Aksium	£42.48	245	N/A	Depor Village
21	Specialized	Echelon MIPS	£63.00	340	MIPS	Sigma Sports
22	Overade	Plixi	£89.99	440	Foldable	Currys
23	Giro	Agilis MIPS	£89.99	300	MIPS	Wiggle
24	Bell	Formula LED MIPS	£89.00	351	Integrated LED, MIPS	Tredz
25	Bontrager	Specter WaveCel	£77.00	360	WaveCel	Sigma Sports
26	Kask	Mojito 3	£83.00	239	N/A	Sigma Sports
27	Bontrager	Velocis MIPS	£99.00	284	MIPS	Sigma Sports
28	Giro	Synthe II MIPS	£80.00	268	MIPS	Wiggle
29	ABUS	GameChanger	£152.00	265	Aero	Sigma Sports
30	Kask	Protone	£135.00	230	N/A	Wiggle
–	Specialized	Tactic MIPS	£85.00	380	MIPS	Sigma Sports

^a^Unavailable at the time of helmet purchase, replaced with Specialized Tactic MIPS (listed in the last row).

### Statistical Analysis

The mean value, averaged across all repeats for a given helmet, were calculated as well as the standard deviation and coefficient of variation (CV). The CV was used to assess helmet test variability across all kinematic metrics. To directly compare a cohort of helmets composed of the same helmets with and without MIPS, the Mann-Whitney *U* test was used due to its ability to account for non-parametric data.

Ordinary least square (OLS) linear models from the Python package ‘statsmodels’ were used to investigate the influence of different factors on injury risk [66]. Three separate OLS models were considered. The first OLS model investigates the influence of impact location, mass, price, presence of MIPS, and distinct helmet model on the location-specific overall risk of injury associated with each test repeat, referred to previously as $P\left(\text{injury }@ \text{location}\right)$. This enables the influence of impact location to be understood in conjunction with other listed factors. The input data included all test repeats collected from 360 tests (all helmets, impact locations and repeats). The mean overall risk at a specific location was used as a baseline for the inter-helmet comparison. In order to compare the influence of impact location, the non-exposure-weighted overall risk was used. The pXR impact location was selected as the baseline impact location as it occurs most frequently in real-world impacts [3]. The second OLS model investigates the influence of almost the same parameters (only impact location is necessarily excluded) on the helmet-specific risk, referred to earlier as $P\left(\text{injury }@\text{ helmet}\right)$. The third OLS model investigates how individual parameters (e.g., purchase price and mass) are affected by linear and/or rotational motion. The helmet mass was taken from online vendors of the helmets. In all instances, we ensure that the assumptions of OLS models (linearity, no multicollinearity, no autocorrelation, homoscedasticity, and a normal distribution of errors) are upheld via the Omnibus and Jarque-Bera tests [67–70].

## Results

### Overview of the Head Kinematics

**Fig. 2:**
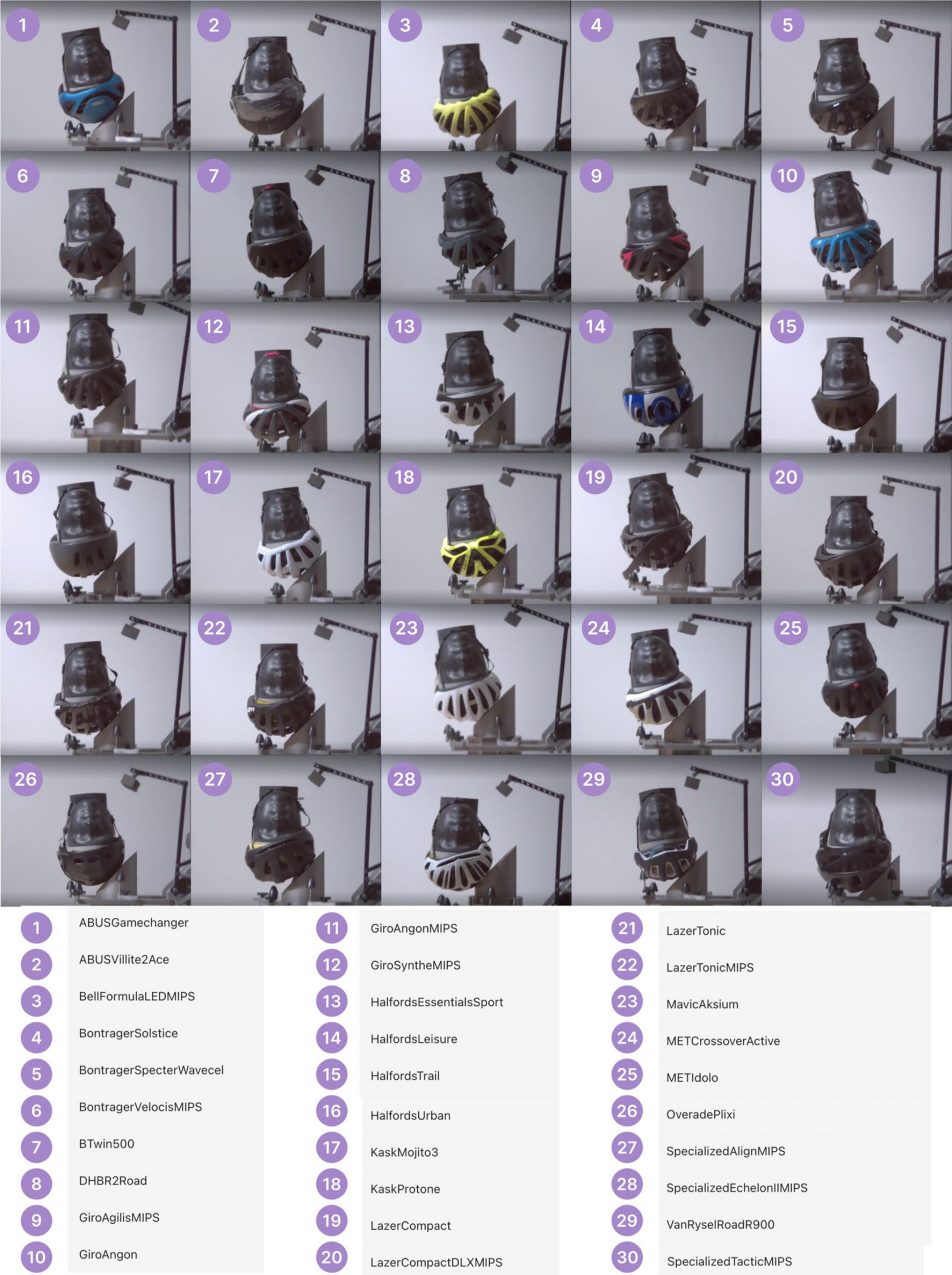
High-speed footage showing helmet response to impact at 20 ms following the helmet/anvil contact initiation. The pXR configuration is shown

Figure 2 shows a snapshot from high-speed footage at 20 ms following the helmet/anvil contact initiation. The headform rotation about the *x*-axis at this time point is noticeable. This figure shows the difference in headform rotation across different helmets, e.g., a large rotation of the headform fitted with helmet 16 compared with helmet 27.

**Fig. 3:**
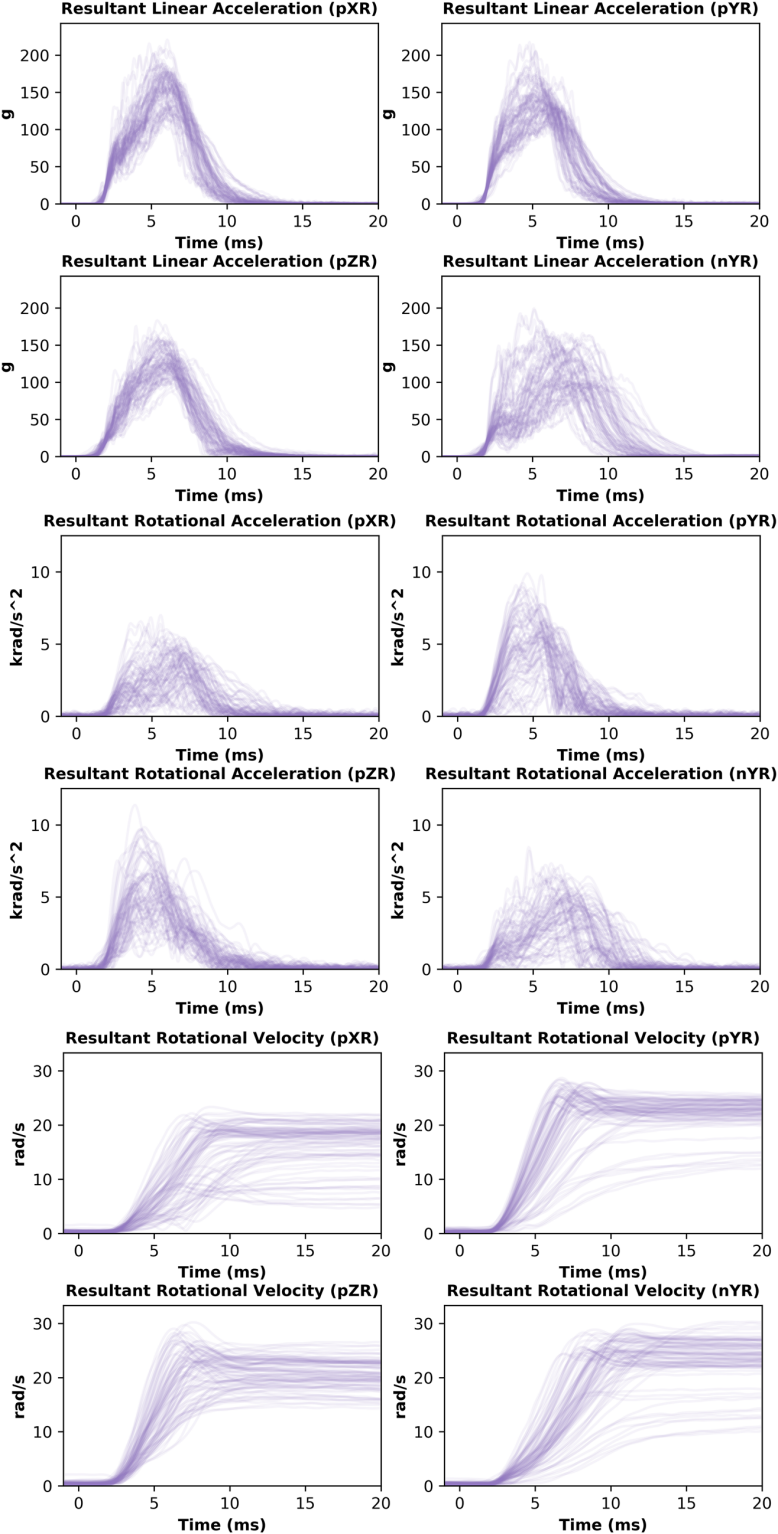
Time-history of head kinematics, showing resultant linear acceleration, resultant rotational acceleration and resultant rotational velocity for all helmets and impact locations

The head kinematic distributions of the whole tested helmet population for all impact locations and repeats are shown in Fig. 3. Each low transparency line represents one helmet drop. The variation in the headform kinematics can be seen by the faded distributions. This figure shows that the duration of impacts is 10–15 ms. It also shows some trends across impact locations. For instance, larger variation in linear acceleration can be seen in nYR impacts than other impact locations. In addition, when looking at the rotational velocity curves, the results are more homogenous for the pZR impact than the other three impact locations. This figure shows that some helmets produce distinctly lower rotational velocities than the rest of the helmets in pYR, pXR, and nYR impacts.

### Good Repeatability of the Tests

**Fig. 4:**
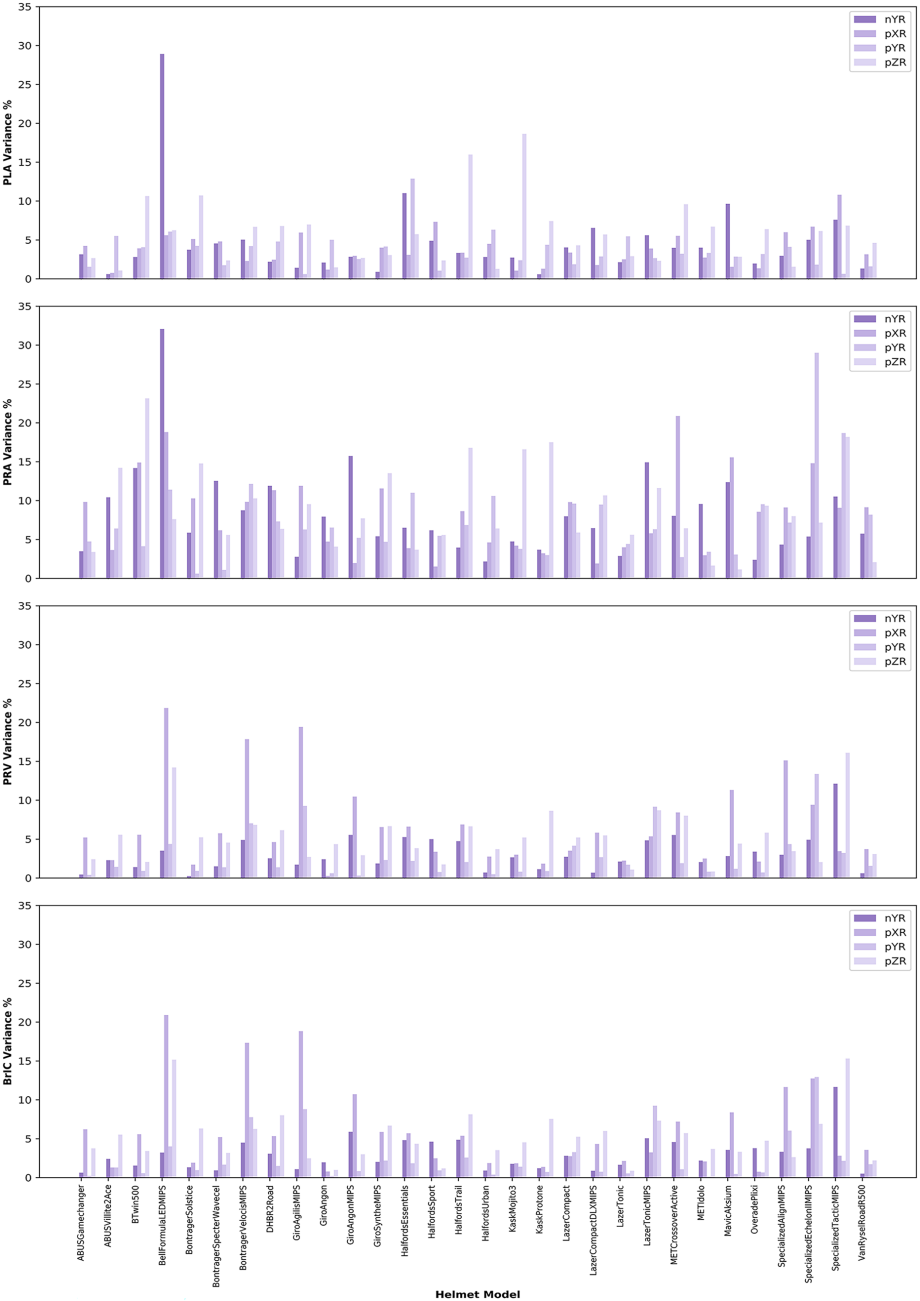
The coefficient of variation of head kinematics metrics for all helmets and impact locations

The coefficient of variation (CV) was below 10% for the majority of helmets, impact locations, and kinematic metrics (Fig. 4). 86.9% (417/480) of CVs across all metrics were below 10%, which includes 93.3% of PLA CVs, 91.7% of PRV and BrIC CVs, and 70.8% of PRA CVs. 98.5% (473/480) of CVs across all metrics were below 20%. The CV is generally higher for PRA than other metrics [CV PLA < CV PRA: *U* = 3483.0, *p* < 0.000; CV PRV < CV PRA: *U* = 3511.0, *p* < 0.0001; CV BrIC < CV PRA: *U* = 3328.0, *p* < 0.0001]. Except for a few helmets and impact locations, the CV for the other metrics was low and does not differ significantly between any other kinematics metrics, showing the good test repeatability.

Different test locations had different CV distributions. In general, nYR and pYR tests had lower CV across all metrics, while pXR and pZR were higher. For all metrics, pYR had the lowest variability [pYR < pXR: *U* = 4905.0, *p* < 0.0001; pYR < pZR: *U* = 4414.0, *p* < 0.0001; pYR < nYR: *U* = 6213.0, *p* = 0.0333]. In addition, nYR variability was lower when compared to pXR and pZR [nYR < pXR: *U* = 5710.0, *p* = 0.0028; nYR < pZR *U* = 5040.0, *p* < 0.0001]. When using a linear model to investigate the interaction between impact location and kinematic metric on CV, the pXR and pZR tests in addition to PRA increased the CV [location = pXR, *t* = 2.629, *p* < 0.001; location = pZR, *t* = 2.897, *p* = 0.004; metric = PRA, *t* = 7.448, *p* < 0.001]. A full summary can be found in “Appendix 2”.

### Head Kinematics Metrics and Injury Risks for Different Helmets

**Fig. 5:**
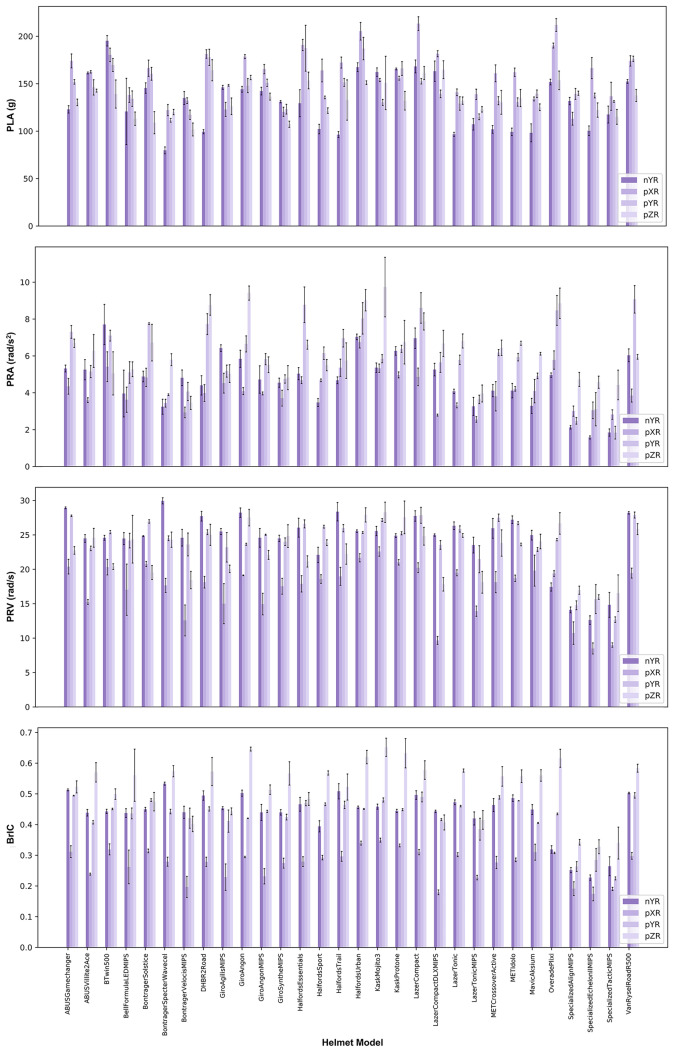
The head kinematics metrics for all helmets and impact locations; the mean value and standard deviation are shown

**Fig. 6:**
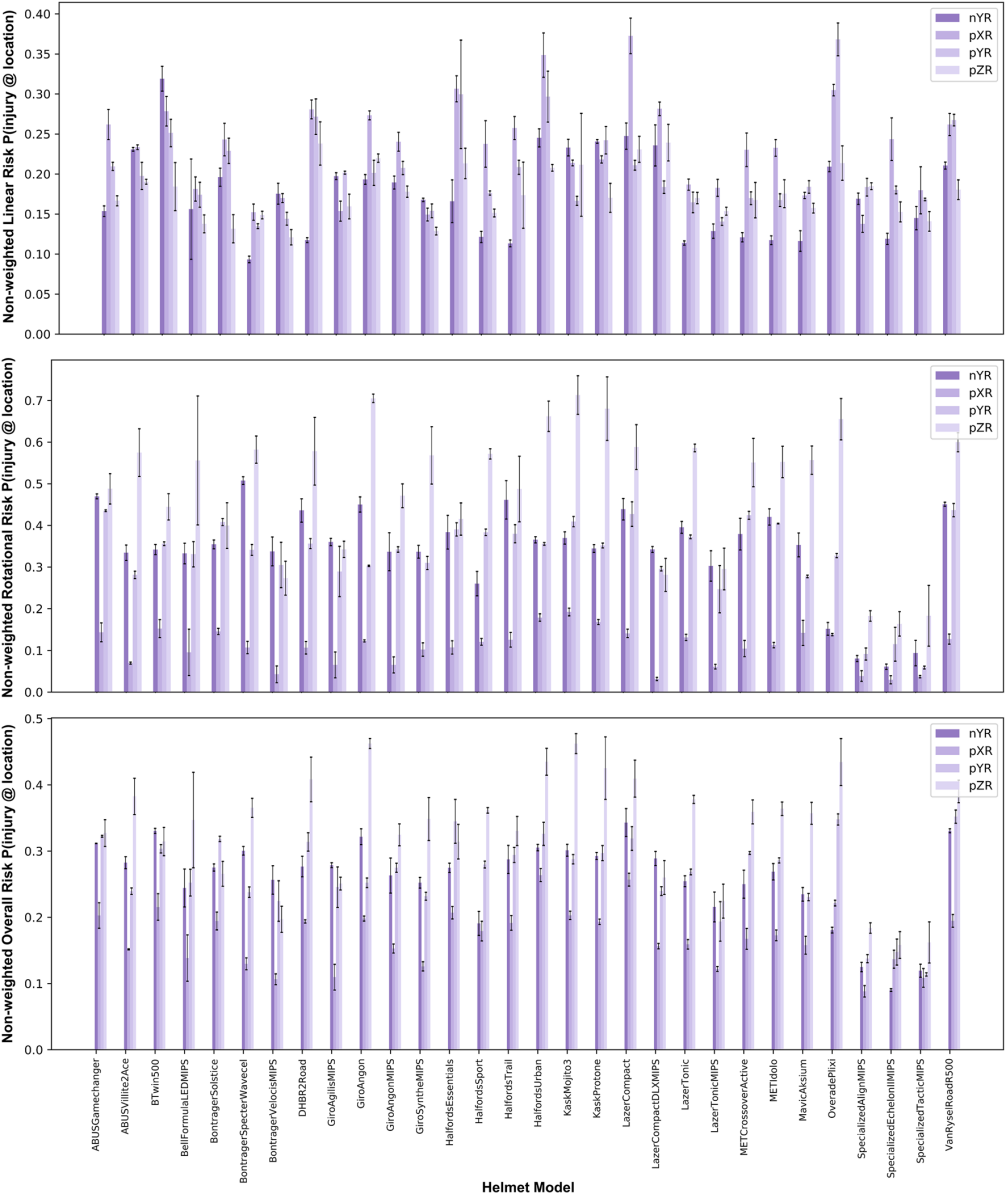
The head injury risks for all helmets and impact locations. The linear risk is based on PLA, the rotational risk is based on BrIC, and the overall risk is the average of these risks

The head kinematics metrics and injury risks vary across the different helmets and impact locations, as shown in Figs. 5 and 6. The ranges of head kinematics and injury risks are provided in Table 5 for each impact location and across all locations, showing large differences between lowest and highest values recorded across all kinematics metrics and injury risks.

**Table 5:** Ranges of kinematic values and three risk metrics associated with different impact locations for the 30 tested helmets

Impact location	PLA (g)	PRV (rad/s)	PRA (rad/s^2^)	BrIC	Linear risk	Rotational risk	Overall risk
pXR	113.1–213.3	8.5–22.6	2.55–6.74	0.174–0.349	0.138–0.373	0.030–0.192	0.088–0.264
pYR	111.4–211.8	14.8–27.9	1.84–9.07	0.225–0.495	0.135–0.368	0.059–0.437	0.114–0.352
nYR	79.7–195.2	12.6–29.9	1.58–7.70	0.227–0.533	0.093–0.139	0.061–0.508	0.090–0.343
pZR	101.7–164.7	16.0–28.3	3.45–9.73	0.328–0.652	0.121–0.239	0.164–0.825	0.158–0.518
All	79.7–213.3	8.5–29.9	1.58–9.73	0.174–0.652	^1^0.093–0.373 ^2^0.135–0.246	^1^0.030–0.825 ^2^0.074–0.326	^1^0.088–0.518 ^2^0.108–0.283

^1^Highest and lowest values of all tests, not accounting for impact location exposure $P\left(\text{injury }@\text{ location}\right)$, shown in Fig. 6.

^2^Highest and lowest values across all helmets, calculated as a weighted sum to account for impact location exposure $P\left(\text{injury }@\text{ helmet}\right)$ not shown in Fig. 6.

### Distinct Effect of Impact Location on Head Kinematic Metrics and Injury Risks

**Fig. 7:**
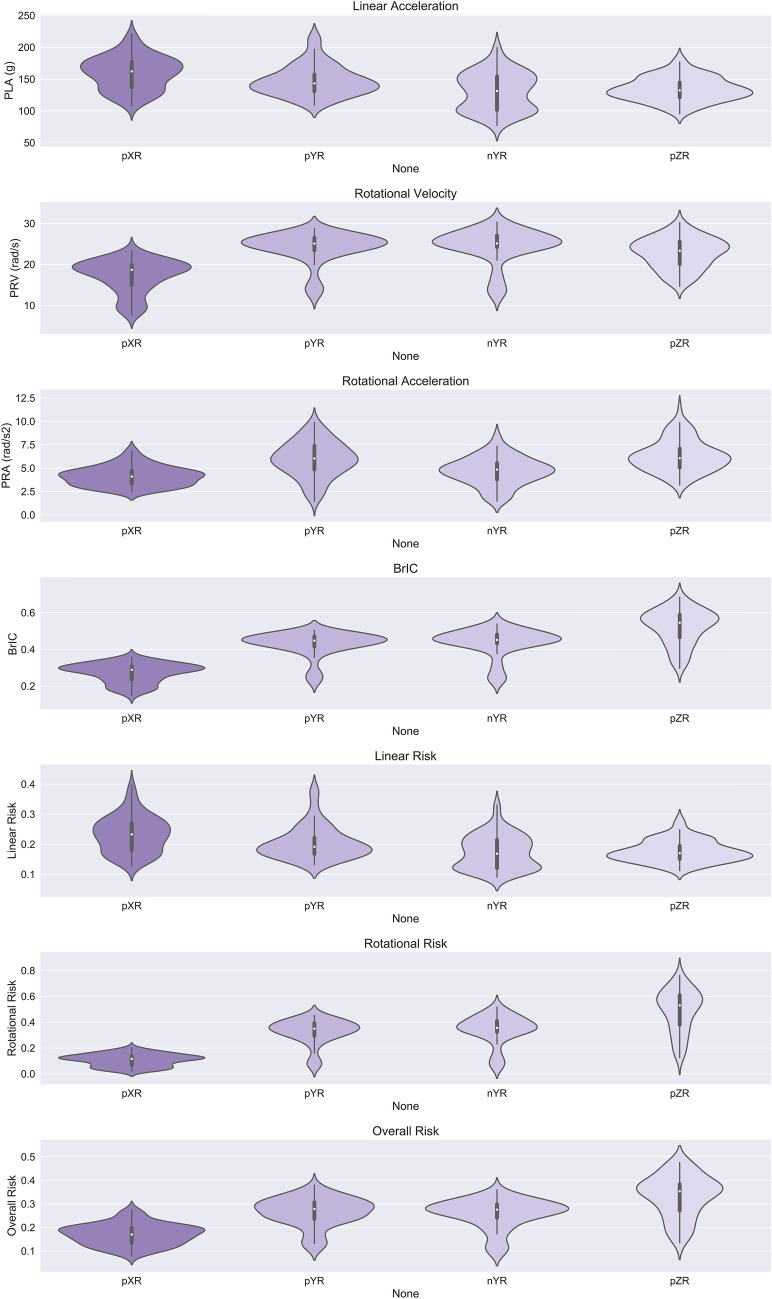
The distributions of head kinematics and risk metrics separated by impact location. The risks shown are not exposure-weighted and follow the definition of $\text{P}\left(\text{injury }@\text{ location}\right)$ set out in the method section

The mean PLA across all helmets and repeats was highest in the pXR impact location (159.9 g) than other impact locations (pYR: 147.7 g, pZR: 134.3 g, nYR: 131.1 g) (Fig. 7). The PLA across all tests in the pXR impact location was significantly higher than the tests in the other three impact locations [pXR > pYR: *U* = 5255.0, *p* = 0.0003; pXR > pZR: *U* = 6299.0, *p* < 0.0001; pXR > nYR: *U* = 6184.0, *p* < 0.0001]. Additionally, the PLA in the pYR impact location was significantly higher than in the nYR and pZR locations [nYR < pYR: *U* = 2857.0, *p* = 0.0003; pZR < pYR: *U* = 2761.0, *p* = 0.0001].

The mean PRA across all helmets and repeats was highest in the pZR (6.31 krad/s^2^) impact location, followed by the pYR (5.94 krad/s^2^), nYR (4.69 krad/s^2^), and finally lowest in the pXR impact location (4.15 krad/s^2^). Statistically, the PRA across all tests at pXR was lower when compared to test conducted at the other three impact locations [pXR < pYR: *U* = 1630.0, *p* < 0.0001; pXR < pZR: *U* = 1054.0, *p* < 0.0001; pXR < nYR: *U* = 2975.0, *p* = 0.0010]. The PRA of the nYR tests was lower than for the pZR and the pYR tests [nYR < pZR: *U* = 6131.0, *p* < 0.0001; nYR < pYR: *U* = 2407.0, *p* < 0.0001].

The mean PRV for the nYR location was highest (24.4 rad/s), followed by pYR (24.1 rad/s), pZR (22.8 rad/s) and finally pXR (17.2 rad/s). Statistically, the PRV across all repeats and helmets was significantly lower for pXR than the other three impact locations [pXR < pYR: *U* = 686.0, *p* < 0.0001; pXR < pZR: *U* = 1263.0, *p* < 0.0001; pXR < nYR: *U* = 832.0, *p* < 0.0001]. The PRV was lower across all tests at pZR than the nYR and pYR locations [pZR < nYR: *U* = 2761.0, *p* = 0.0001; pZR < pYR: *U* = 2958.0, *p* = 0.0008].

The mean BrIC across all helmets and repeats was highest in the pZR (0.523) impact location, followed by the pYR (0.450), nYR (0.436), and finally lowest in the pXR impact location (0.272). When comparing all tests at a given impact location statistically, pZR tests produced higher BrIC across all tests [pZR > nYR: *U* = 6254.0, *p* < 0.0001; pZR > pYR: *U* = 6434.0, *p* < 0.0001; pXR < pZR: *U* = 91.0, *p* < 0.0001].

Since the linear and rotational injury risks are monotonic functions of PLA and BrIC, respectively, the impact location has the same effect on these risks as it has on PLA and BrIC. Most notably, the largest linear risk was seen in pXR impacts [pXR > pYR: *U* = 5255.0, *p* = 0.0003; pXR > pZR: *U* = 6299.0, *p* < 0.0001; pXR > nYR: *U* = 6184.0, *p* < 0.0001] (Fig. 7). The largest rotational risk was seen in pZR impacts [pZR > nYR: *U* = 6254.0, *p* < 0.0001; pZR > pYR: *U* = 6434.0, *p* < 0.0001; pZR > pXR: *U* = 91.0, *p* < 0.0001]. The largest overall risk was seen in pZR impacts [pZR > nYR: *U* = 6215.0, *p* < 0.0001; pZR > pYR: *U* = 6079.0, *p* < 0.0001; pZR > pXR: *U* = 563.0, *p* < 0.0001].

The OLS model built to investigate the influence of impact location, helmet type, mass, price, and presence of MIPS on non-exposure-adjusted overall risk, $P\left(\text{injury}@\text{location}\right)$, confirmed that pYR, nYR, and pZR all differed significantly from pXR. Significantly higher overall risk was seen in pYR [coefficient: 0.0960, *t* = 16.7, *p* < 0.001], nYR [coefficient: 0.0883, *t* = 15.3, *p* < 0.001] and pZR [coefficient: 0.1635, *t* = 16.7, *p* < 0.001] when compared to the pXR baseline. The coefficients show that the overall risk is approximately 0.1 higher for pYR and nYR compared to pXR, and 0.17 higher for pZR (the location with the highest associated risk). The findings related to other OLS model parameters are detailed within the relevant sections.

### Exposure Weighted Linear, Rotational, and Overall Injury Risk: Ranking of Helmets

**Fig. 8:**
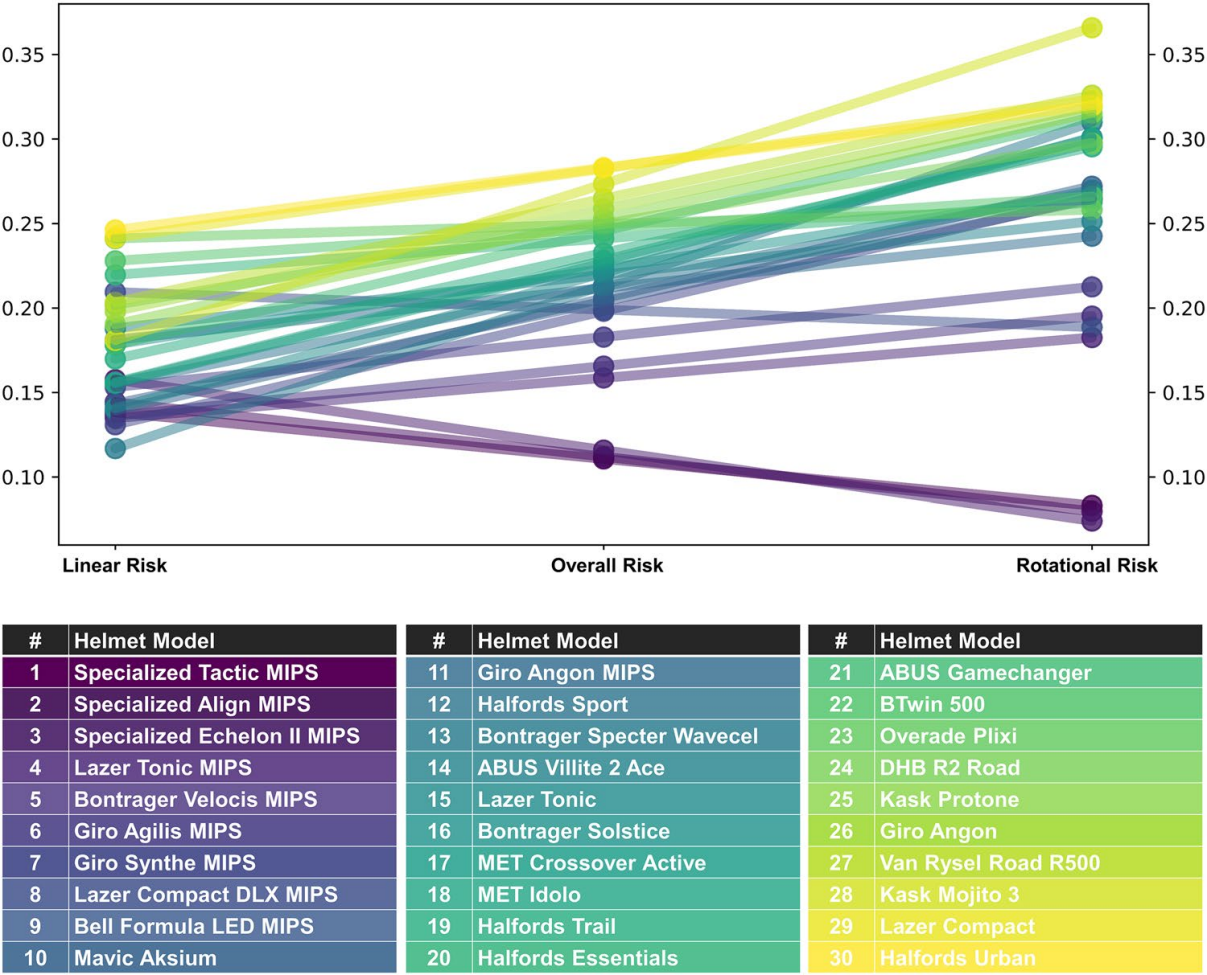
A visualization of the influence of linear and rotational risk on the overall risk rank and values. The first subplot shows (from left to right) linear, overall, and rotational risk ranks while the second subplot shows (from left to right) linear, overall, and rotational risk values including their distributions. The colored table at the base acts as a color-coded reference to the helmets on the plot

Finally, we used the impact location weighting to calculate one value for the linear, rotational, and overall risk for each helmet type, $P(\text{injury}@\text{helmet})$ and rank them based on the overall risk (Table 6). We observed a larger variation in rotational risk than the linear risk, as shown in Fig. 8. The worst performing helmet of the 30 cohort (rank #30) had a 2.62 times higher overall injury risk compared to the best performing helmet (rank #1). When considering the linear and rotational components of the overall worst and best performing helmets, this ratio was 1.76 for the linear risk and 4.21 for the rotational risk.

**Table 6:** The overall, linear, and rotational injury risk (mean value of the three repeats) and overall rank of the helmets are shown with the purchase price (2022–23) in GBP and mass in grams, where overall risk was calculated as an average of the linear and the rotational risk

Overall rank	Helmet	Overall risk	Linear risk	Rotational risk	Purchase price (£)	Mass (g)
1	Specialized Tactic MIPS	0.108	0.140	0.076	£50.00	380
2	Specialized Align MIPS	0.112	0.144	0.080	£34.00	355
3	Specialized EchelonII MIPS	0.116	0.158	0.074	£63.00	340
4	Lazer Tonic MIPS	0.159	0.135	0.182	£65.00	230
5	Bontrager Velocis MIPS	0.163	0.135	0.191	£99.00	284
6	Giro Agilis MIPS	0.183	0.153	0.212	£89.99	300
7	Giro Synthe MIPS	0.198	0.131	0.265	£100.00	268
8	Lazer Compact DLX MIPS	0.199	0.209	0.189	£54.99	325
9	Bell Formula LED MIPS	0.204	0.143	0.264	£77.99	351
10	Mavic Aksium	0.205	0.139	0.272	£42.48	245
11	Giro Angon MIPS	0.212	0.181	0.242	£79.99	280
12	Halfords Sport	0.213	0.155	0.270	£15.00	240
13	Bontrager Specter Wavecel	0.213	0.117	0.310	£62.00	360
14	ABUS Villite 2 Ace	0.220	0.188	0.251	£89.99	400
15	Lazer Tonic	0.221	0.141	0.301	£40.00	230
16	Bontrager Solstice	0.223	0.178	0.268	£14.00	310
17	MET Crossover Active	0.224	0.155	0.293	£22.50	295
18	MET Idolo	0.228	0.156	0.300	£25.00	255
19	Halfords Trail	0.233	0.170	0.295	£25.00	280
20	Halfords Essentials	0.241	0.220	0.263	£10.00	240
21	ABUS Gamechanger	0.246	0.178	0.313	£127.00	265
22	BTwin 500	0.247	0.228	0.266	£9.99	560
23	Overade Plixi	0.250	0.241	0.258	£82.82	440
24	DHB R2 Road	0.250	0.202	0.298	£25.00	273
25	Kask Protone	0.253	0.190	0.315	£135.00	230
26	Giro Angon	0.259	0.198	0.319	£64.99	280
27	Kask Mojito 3	0.263	0.181	0.345	£99.00	239
28	Van Rysel Road R900	0.265	0.203	0.326	£29.99	275
29	Lazer Compact	0.283	0.242	0.323	£40.00	325
30	Halfords Urban	0.283	0.246	0.320	£25.00	450
–	Mean	0.216	0.175	0.256	£56.62	310

Of the 9 helmets which had rotational risk lower than the mean rotational risk of 0.256, 6 (66.7%) were also below the mean linear risk of 0.175, including the 6 helmets with the lowest rotational and overall risks. However, of the 15 helmets that had linear risk lower than the mean linear risk of 0.175, only 6 (40%) were also below the mean rotational risk of 0.257. A single variate OLS model predicting rotational risk using linear risk suggests there is a trend toward lower rotational risk being associated with lower linear risk, significant at *p* = 0.10 level but not *p* = 0.05 level [coefficien* t* = 0.173, *t* = 2.0, *p* = 0.055].

### MIPS Reduces Rotational Kinematics and Risk

Ten of the top eleven most protective helmets in terms of overall risk had the MIPS add-on technology (Table 6). MIPS was a factor included in the non-exposure-weighted OLS model that also included helmet model, price, and mass. The presence of MIPS reduced overall non-location weighted risk by 0.0729 [*t* = − 21.3, *p* < 0.001].

To investigate whether MIPS was causal in this protective effect, we compared the three helmets which had models with and without MIPS using Mann–Whitney U tests (Table 7). Across all 72 tests conducted at different impact locations for the three helmets with and without MIPS, MIPS significantly reduced PRV by 19% [*U* = 263.0, *p* < 0.0001], PRA by 27.6% [*U* = 302.0, *p* < 0.0001], and BrIC by 18.5% [*U* = 336.0, *p* = 0.0002] but not PLA [*U* = 545.0, *p* = 0.1242]. The significance holds when considering the helmet models with and without MIPS individually. The Giro Angon MIPS had significantly lower PRV [*U* = 42.0, *p* = 0.044] and PRA [*U* = 37.0, *p* = 0.023] but not BrIC [*U* = 58.0, *p* = 0.218] or PLA [*U* = 47.0, *p* = 0.079] compared to the Giro Angon (without MIPS). The Lazer Tonic MIPS had significantly lower PRV [*U* = 21.0, *p* = 0.002], PRA [*U* = 26.0, *p* = 0.004], and BrIC [*U* = 27.0, *p* = 0.005] but not PLA [*U* = 57.0, *p* = 0.201] compared to the Lazer Tonic (without MIPS). The Lazer Compact with MIPS demonstrated significantly lower PRV [*U* = 25.0, *p* = 0.004], PRA [*U* = 29.0, *p* = 0.007], and BrIC [*U* = 27.0, *p* = 0.005] but not PLA [*U* = 61.0, *p* = 0.272].

**Table 7:** A comparison of the effect of MIPS technology on three helmet models tested with and without MIPS is presented with mean kinematics (PLA, PRV, PRA, and BrIC) and risk (linear, rotational, overall) values and associated statistics across all test repeats and impact locations (*n* = 72), with the differences between the MIPS and no-MIPS values (indicated by a ‘−’ in the MIPS column)

Metric	With MIPS (*n* = 36 tests)	Without MIPS (*n* = 36 tests)	% difference of adding MIPS
PLA (g)^1^	144.0	151.7	− 5.1% [*U* = 545.0, *p* = 0.1241]
PRA (rad/s^2^)^1^	4.48	6.19	− 27.6% [*U* = 302.0, * p* < 0.0001]
PRV (rad/s)^1^	20.0	24.6	− 18.7% [*U* = 263.0, * p* < 0.0001]
BrIC^1^	0.377	0.462	− 18.4% [*U* = 336.0, * p* = 0.0002]
Linear Risk^2^	0.1751	0.1934	− 9.5% [*U* = 29.0, * p* = 0.1657]
Rotational Risk^2^	0.2045	0.3146	− 35.0% [*U* = 0.0, * p* = 0.0002]
Overall Risk^2^	0.1898	0.2540	− 25.3% [*U* = 1.0, * p* = 0.0003]

^1^Calculated as a mean across all 36 tests with MIPS and all 36 tests without MIPS

^2^Calculated as a mean after exposure-weighted risk was calculated across all 4 test locations, leaving 9 repeats with and without MIPS

Linear, rotational and overall risk were also compared across the three helmets which had MIPS and no-MIPS versions. Helmets with MIPS significantly reduced the overall risk by 33.8% [*U* = 1.0, *p* = 0.0003] and rotational risk by 53.8% [*U* = 0.0, *p* = 0.0002] but not the linear risk [*U* = 29.0, *p* = 0.1657]. When comparing individual helmets, the overall and rotational risks were reduced for the Giro Angon, Lazer Compact, and Lazer Tonic models when MIPS was included [all *U* = 0.0, *p* = 0.0404]. The linear risk was reduced for the Lazer Compact and Giro Angon [both *U* = 0.0, *p* = 0.0404], but not the Lazer Tonic [*U* = 2.0, *p* = 0.1914].

### No Influence of Price on Protection

**Fig. 9:**
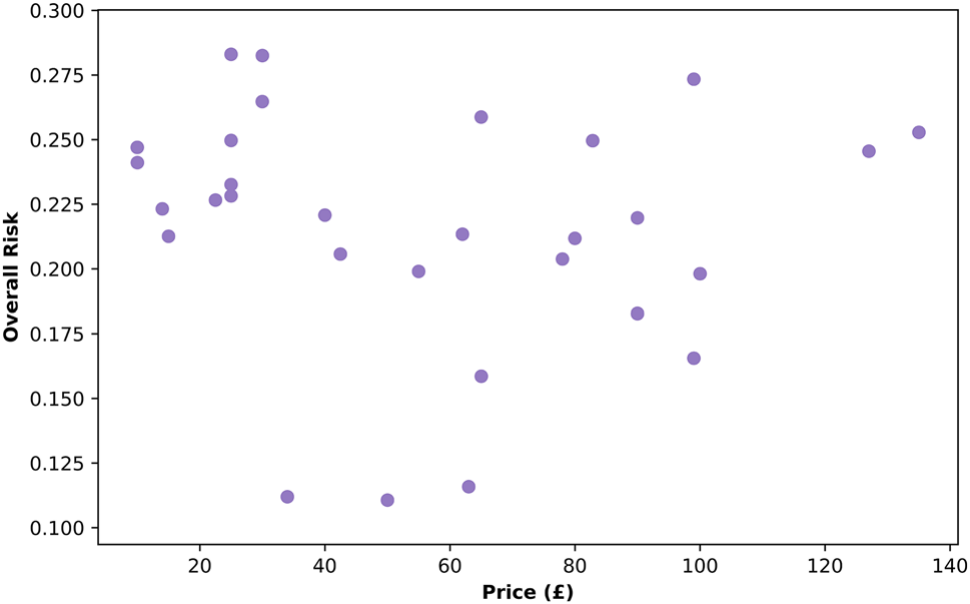
A scatter plot showing helmet purchase price vs overall risk for all thirty tested helmets

The price of helmets in our cohort varied between £9.99 and £135.00 (GBP). A visual summary of price vs linear and rotational risk does not indicate any association between them (Fig. 9). This was confirmed using the OLS models. Predicting overall, linear and rotational risk from price showed that there was not a significant influence of price on protection [overall risk: *p* = 0.755; linear risk: *p* = 0.263; rotational risk: *p* = 0.799]. The same conclusions were reached when considering all three risk values in conjunction to predict price [overall risk: *p* = 0.755; linear risk: *p* = 0.790; rotational risk: *p* = 0.747].

This differed slightly when considering non-exposure-weighted risk. The OLS model combining helmet model, impact location, presence of MIPS, price, and mass showed that price had a small influence on non-exposure-weighted location-specific overall risk. For every 1GBP increase in price, the overall non-location-weighted risk increased by 0.0007 [*t* = 7.6, *p* < 0.001]. The difference between the cheapest (£10) and most expensive helmet (£135) in the sample is £125, which based on the OLS output corresponds to a difference of 0.0875 in risk, with more expensive helmets associated with a higher risk. Note that it was not possible to repeat this analysis for overall risk (adjusted for exposure) due to the assumptions of OLS modeling not being upheld in that instance. Details of this model can be found in “Appendix 3”.

### Mass of Helmet Significantly Affects Linear Protection

**Fig. 10:**
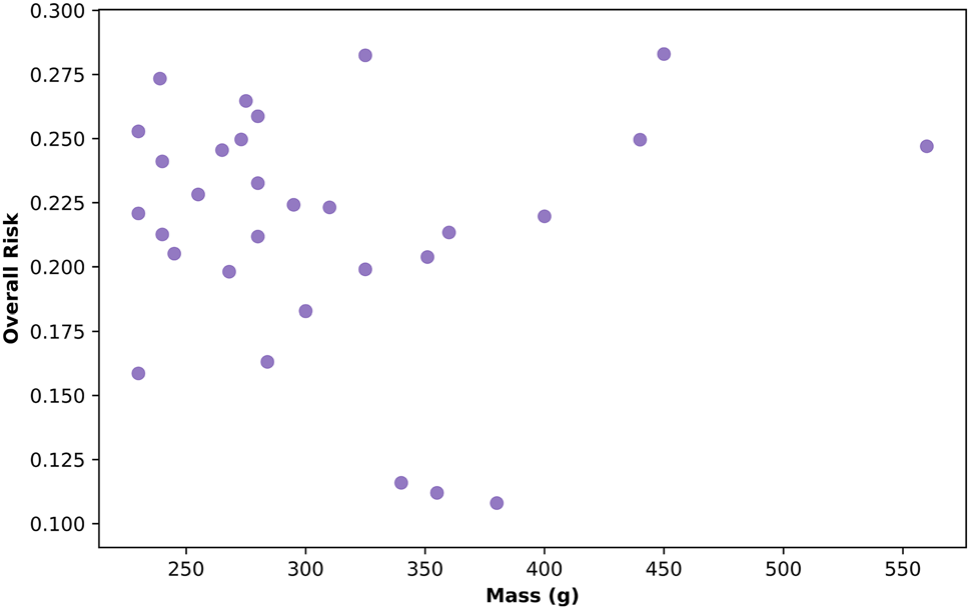
A scatter plot showing the mass of the helmet in grams (taken from vendor or manufacturer websites) vs the overall risk for all thirty tested helmets

Helmet masses in our cohort varied between 230 and 560 g. A visual summary of mass vs overall risk is shown in Fig. 10. In single variate OLS models, we observed no significant effect of mass on rotational risk [*t* = − 1.135, *p* = 0.266] or on overall risk [*t* = − 0.060, *p* = 0.952], which can also be seen in Fig. 10. However, there was a relationship between linear risk and mass [*t* = 2.307, 0.029], inferring that heavier helmets were associated with higher linear risk. An OLS model which assessed the combined effect of linear and rotational risk in relation to mass showed that mass depended on both the rotational risk and linear risk, in different directions [rotational risk can predict mass: *t* = − 2.297, *p* = 0.030; linear risk can predict mass: *t* = 3.118, *p* = 0.004]. This infers that increasing rotational risk was associated with decreasing mass and conversely, that increasing linear risk is associated with increasing mass.

When considering non-exposure-weighted overall risk, the OLS model combining helmet model, impact location, presence of MIPS, price, and mass showed that mass had a small influence on non-weighted location-specific overall risk. For every gram increase in mass, the overall non-location weighted risk increased by 0.0004 [*t* = 6.9, *p* < 0.001]. The 330 g difference between the lightest and heaviest helmet corresponds to a difference of 0.132 non-location adjusted overall risk, with heavier helmets associated with higher risk. Note that it was not possible to repeat this analysis for overall risk (adjusted for exposure) due to the assumptions of OLS modeling not being upheld in that instance.

## Discussion

We presented the protection performance of 30 most popular bicycle helmets against skull fractures associated with the head linear motion and diffuse brain injuries associated with the head rotational motion. We used the new biofidelic headform for testing the helmets, allowing us to predict head linear and rotational responses more accurately [37]. In addition, we used an evidence-based test protocol enabling to assess performance under representative impact conditions [3]. Head kinematics and overall, linear, and rotational injury risks varied substantially across helmets, demonstrating that there are large differences in their protection, although all helmets had passed the standard impact tests of the EN1078 standard. Interestingly, we observed a greater difference in rotational compared to linear risk protection. The ratio between the highest and lowest rotational risk was over two folds the ratio between the highest and lowest linear risk. This suggests that the helmets tested are better optimized for managing head linear motion than rotational motion. This is likely due to the absence of rotational testing in helmet standards to date, an area that needs to be addressed in future standards in order to improve the protection of helmets against injuries caused by head rotation, particularly diffuse brain injuries.

MIPS was equipped to the top 9 and 11th overall most protective helmets, 8 of which were helmets with the lowest rotational risk (0.074–0.242). MIPS has been shown to be effective in reducing rotational motion when testing with the Hybrid III (HIII) headform across a range of headform surface conditions, including bare, with a stocking to reduce the coefficient of friction and with hair [71]. This study is the first to show that MIPS remains effective at mitigating rotational motion with the new headform that is more biofidelic than the HIII headform in terms of two key factors affecting head rotation, the coefficient of friction and moments of inertia [37, 72, 73]. Importantly, in the direct comparison between matched helmet models with and without MIPS, we found that linear acceleration was not significantly reduced by MIPS. While MIPS drives overall risk down by reducing rotational risk, it does not drive a reduction in linear risk. Interestingly, the helmet with the Wavecel technology, designed for rotational risk mitigation [74], produced the lowest linear risk (0.141) in our cohort of 30 helmets, although its overall rank was #13 due to the high rotational risk (0.301). These results show that it is vital to design helmets holistically to reduce both linear and rotational kinematics metrics to protect against a range of different head injury types caused by different mechanisms.

We observed a distinct effect of impact location on head kinematics and subsequently the linear and rotational risks calculated from PLA and BrIC, respectively. The largest PLA and linear risk were seen in pXR impacts. The largest BrIC and rotational risks were seen in pZR impacts. Interestingly although both BrIC and PRV are driven from head rotational velocity, the largest PRV was produced in nYR impacts, rather than pZR. This is because BrIC has a lower critical angular velocity for the *z* axis rotation than *x* and *y*, i.e., it exaggerates the effects of rotation about the *z* axis. A recent study of eight established brain FE models using kinematics data from cycle helmet oblique impacts has shown stronger correlation between BrIC and brain strain, than PRV [61]. This further supports adopting BrIC for assessing the effects of head rotational motion on the brain in our helmet impacts. Our findings show the particular importance of the pXR and pZR impact locations in producing highest linear and rotational risks, respectively, which should be a target for improving helmet design.

Although we found that there is no relationship between helmet price and the linear, rotational, or overall risk in isolation, our OLS model of non-exposure-weighted overall risk based on helmet model, mass, price, and presence of MIPS suggested an effect of increased price on increased risk. The lack of relationship in the isolated models is in keeping with previous work that has shown a weak negative correlation between cycle helmet price and risk of “concussion” measured under oblique impacts [33, 75]. The lack of relationship between price and safety performance is important because in the absence of objective information about helmet safety, consumers may rely on the price to indicate the safety performance [40, 41]. Previous work has shown that higher likelihood of helmet use is correlated with higher income and employment status [76]. Consumers should be able to access clear information about the protection performance of helmets, particularly relating to more affordable options.

Our survey demonstrated that helmet mass is a factor considered to be important in the road cycling community, likely due to lower mass equating to higher speed and comfort. Of 1083 helmet wearers, 890 (82.2%) found helmet mass to be either “very important,” “important,” or “somewhat important” compared to 193 (17.8%) who found mass to be “not important” or “not important at all.” The results of the multivariate OLS models suggested that increasing rotational risk is associated with decreasing mass and conversely, that increasing linear risk is associated with increasing mass (additionally supported by a single variate model). The fact that heavier helmets were associated with higher linear risk is in contrast to one of our incoming hypotheses that heavier helmets have more material and therefore offer better linear protection due to higher potential for the liner to remove energy in an impact. Our second hypothesis that lighter helmets are product of increased research and development, which is associated with better performance, is one possible reason that lighter helmets offered better linear protection. We found no studies on the relationship between bicycle helmet mass and protection, making this a novelty of our study. In one 1996 study of fatal motorcyclist incidents with axial load shift, heavier motorcycle helmets (> 1500 g among a range of 600–2000 g) were associated with higher basilar skull fracture risk [77]. The authors found no increased risk below 1500 g. Our helmets ranged from 230 to 560 g, making all bicycle helmets we tested lighter than the motorcycle helmet in the previously mentioned study. This previous work additionally assessed risk of a specific injury, whereas we use a combined overall risk metric assessing the risk of diffuse and focal injuries. Importantly, our OLS model shows that increasing mass is associated with increasing overall risk, a finding which may be of interest to the road cycling community who use mass as a purchase factor.

We repeated the tests three times using a new sample. This enabled us to quantify the variation across the tests, which can be attributed to both the test conditions and helmet manufacturing. Such information is missing in previous studies that have assessed helmet performance [32, 33]. The coefficient of variation for the peak kinematic values was found to be below 10% for 87% of tests across all metrics, with 99% below 20%. The variation was highest in the peak rotational acceleration, which is likely due to the fact that PRA was obtained by differentiating the filtered rotational velocity. We chose not to apply an additional filter to the rotational acceleration pulse, as this would act as a smoother when filtering for a second time. The CV for the other kinematic metrics was low and did not differ significantly, further demonstrating consistency of the test conditions and helmet samples. The low CV particularly provides more support for the adoption of the new headform in helmet standards and rating systems that use an isolated headform.

The Cellbond-CEN 2022 headform used in this study has further evolved since this study was completed. The focus of the improvements has been on lowering the CoF to fall within the 0.27–0.33 range, which overlaps with the range of CoF reported for the contact between human head and EPS foam (95% CI static CoF = 0.30–0.34, dynamic CoF = 0.26–0.28) and polyester liner (mean ± STD = 0.29 ± 0.07) [78, 79]. The mean value of the CoF that we measured for the headform was 0.4 ± 0.01, which is 21% higher than the upper value of the target CoF range. A study of cycle helmet oblique impacts using the HIII and NOCSAE headforms has shown that a 55–58% reduction in CoF to around 0.37–0.38 by adding a skull cap to the headforms had a small effect on PLA, PRA and PRV, decreasing them between 2 and 14% [44]. It is likely that using a more recent version of the CEN headform with lower CoF, our results will be affected within a range similar to that reported in this previous study.

Different headforms are currently used in standards and research studies, including EN960, NOCSAE, and HIII, but often their CoF or MoI are not reported or controlled, or they do not agree with human data [44]. Several studies however have investigated helmet response using headforms with more biofidelic MoI or CoF, showing that the headform kinematics is influenced by both [37, 42–44, 73, 80–82]. The new CEN headform is designed with MoI and CoF that better matches those of the human head, addressing the shortcomings of other headforms used for helmet testing. The adoption of this headform by researchers and test labs will enhance the reproducibility of helmet tests.

One novelty of this study is that injury risk functions developed for skull fractures and associated focal injuries and diffuse brain injuries were used, covering a large range of pathologies reported in helmeted cyclists’ incidents. Using a risk function allows for producing one overall risk for a helmet, in contrast to using kinematics values only [32, 83]. Previous studies have used risk functions focused on predicting the risk of “concussion” [33, 39]. Here we provided a more comprehensive picture of helmet protection against a range of injuries. A limitation of this approach is that the risks of linear and rotational injuries are averaged, assuming they have equal importance and presence in real-world casualties. With more data on the distribution of these injuries and their consequences, the weighting of these risks should be adjusted in future.

The rotational risk function is based on BrIC. This metric has had the best correlation with the strain predicted by a range of brain FE models [61]. The BrIC risk function used here was scaled by the developers of BrIC to predict mild diffuse brain injuries, which is a limitation [36]. A recent study has developed a mild traumatic brain injury risk function for BrIC based on data from professional American Football [84]. This function however provides zero risk for small values of BrIC. This study assumed that mild TBI is equivalent to “concussion,” an assumption that has been debated by neurologists, who suggest shifting the focus from symptoms to the likelihood of brain structural damage [85]. Supporting this suggestion, recent work has shown that the count of concussions is not associated with neurodegeneration in American Football players, while cumulative head linear and rotational accelerations can predict neurodegeneration [86]. Hence, in the absence of a better risk function for BrIC, here we used a more conservative risk function that associates a non-zero risk to small values of BrIC.

In this study, only four impact locations are tested, while real-world head impacts can occur in many different locations. We however ensured to select the locations impacted most frequently based on a meta-analysis of 815 cycle helmets [3]. We used the results of this analysis to weight the injury risk associated with each impact location for exposure in contrast to previous work [33, 39]. The exposure was highest in the pXR (side) impact, followed by the nYR (rear) impact and then the pYR and pZR (frontal) [3]. We however did not include a crown impact due to it being the lowest exposure impact location based a meta-analysis of 815 cycle helmets. One further recent study assessing head impact location by soft tissue damage also suggests that crown impacts are uncommon, with soft tissue injuries to the parietal region representing the lowest proportion of soft tissue injuries to the head region (excluding the face), at just 15% [87]. Additionally, all helmets which go to market in Europe and the UK must pass standards, which include impacting the crown region. Therefore, a minimum threshold of protection for impacts to the crown is obtained for all helmets in this cohort.

Consistent use of OLS models was limited due to some datasets violating the assumptions for modeling, in particular non-normal distribution of errors and residuals (identified using the Omnibus and Jarque-Bera tests). This led to an inability to use OLS models to assess the location exposure-adjusted overall risk, $P(\text{injury}@\text{helmet})$.

Another limitation of this study is that we tested medium-sized helmets only, as dictated by the physical headform. Although future development will include a range of sizes for the new headform, at the time this study was conducted, the headform corresponds to an average adult (57 cm circumference based on a height of 175 cm) [88]. Our survey of cyclists showed that on average, males tended to wear medium or large helmets, while females tended to wear small or medium helmets, with a minority of males wearing small helmets and a minority of females wearing large helmets. This corresponds to previous work which demonstrates that head circumference is a function of height, with a slight difference between the sexes, whereby females have a 1.38 cm smaller head circumference at the same height [88]. Although a large proportion of the adult population do wear a medium helmet, future work should ensure that different helmet sizes are tested, promoting equitable research.

We selected the most popular cycle helmets used on the UK roads. This led to the inclusion of two distinct helmet technologies in the cohort of helmets studied here, MIPS and Wavecel. There are several other helmet technologies that are currently available in commercially available helmets [83]. Although these technologies were not within the helmets selected here, they warrant testing according to the protocol used in this study, allowing for a comparison between their performance and the performance of the most popular helmets included in this study. An aim of future work should be to provide the opportunity for the developers of new helmet technologies to submit their helmets for assessment by independent test labs, with the results being published for consumers’ information. This approach helps the designers to better understand the comparative performance of their helmets. It also helps consumers to learn about most protective helmet technologies that may not be popular yet.

In summary, we present the protective performance of 30 bicycle helmets which are popular in the UK under oblique impacts using a new, biofidelic headform and evidence-based test protocol enabling to assess performance under representative impact conditions. Below is a summary of the key findings:

- The least protective helmet had a 2.62 times higher overall head injury risk than the most protective helmet. There was a lesser spread of linear risk (1.76 times) compared to rotational risk (4.21 times).
- The pXR and pZR impact locations produced highest linear and rotational risks, respectively.
- The nine helmets offering the best overall injury protection were all equipped with the anti-rotation technology MIPS, which in direct helmet comparisons between models with MIPS and no-MIPS versions, was shown to be effective in reducing rotational kinematics and risk under the impact conditions tested. However, not all helmets equipped with MIPS were the most protective.
- Mass and price in isolation did not demonstrate a significant effect on exposure-weighted linear, rotational and overall risk, $P(\text{injury}@\text{helmet})$. However, the OLS model predicting non-exposure-weighted overall risk, $P(\text{injury}@\text{location})$, using price, mass, presence of MIPS and impact location showed that both price and mass had a small influence on the non-exposure-weighted overall risk, with increased mass and price shown to relate to increased non-exposure-weighted overall risk.

Our study highlights the need for distinct linear and rotational injuries with different mechanisms to be mitigated through continued improvement in helmet test methods and helmet designs. It also supports the need for providing consumers with objective information about helmet impact performance to help them with choosing most appropriate helmet.

## Figures and Tables

**Fig. 11:**
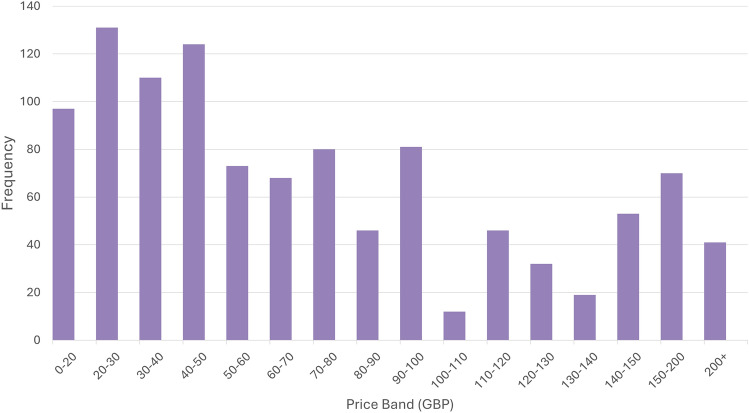
Histogram showing the frequency of helmets across a range of retail price bands (in GBP, determined as of May 2022) of survey responders cycling helmets, based on 1083 who owned a helmet (out of 1132 collected survey responses)

**Fig. 12:**
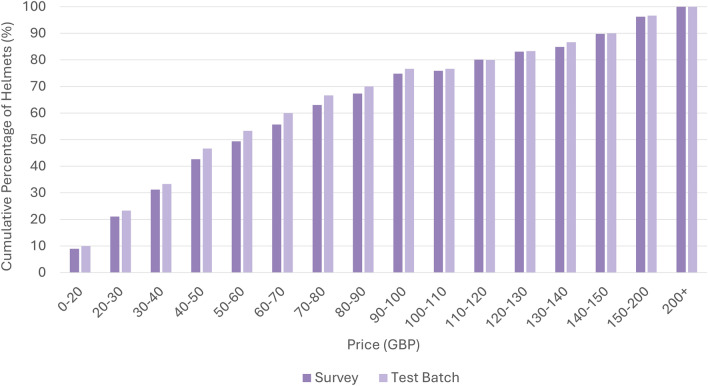
The cumulative price distributions of the 30 helmets selected for testing and the helmet prices obtained via survey responses

**Fig. 13:**
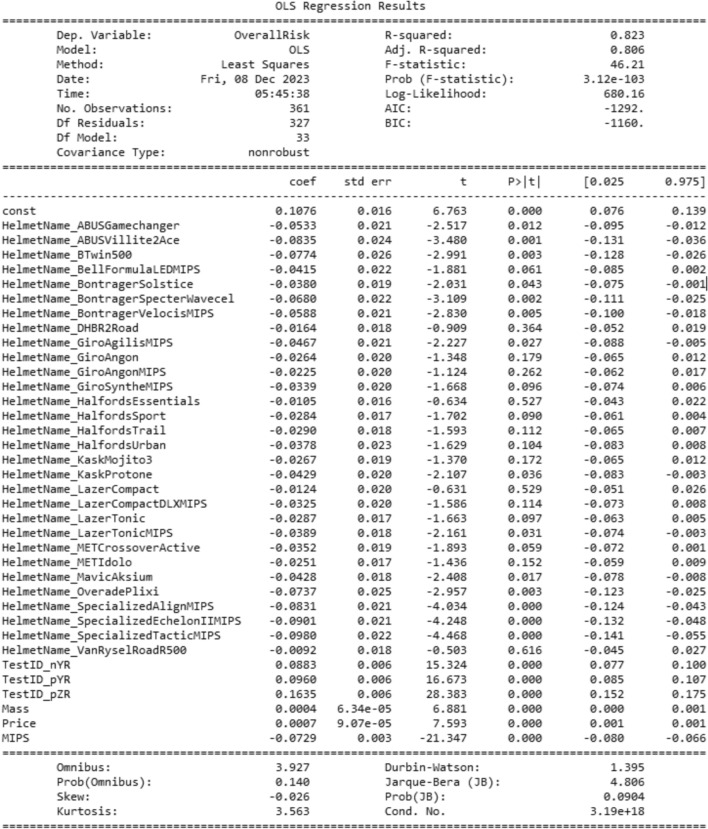
The OLS model summary is shown for the comprehensive model to predict overall risk not adjusted for location exposure, $\text{P}\left(\text{injury}@\text{location}\right).$ Helmet type, impact location, mass, price, and presence of MIPS are used as factors

## Appendix 1

### Helmet Selection and Price Distribution

The information about the make or model of helmet was used to inform the selection of the remaining 17 helmets for testing. Firstly, each unique helmet response was listed (collected from respondents via a free-text field), accounting for discrepancies in spelling and capitalisation, resulting in 214 unique helmets. 20 of these were discontinued and 15 could not be found for purchase, resulting in a list of 179 helmets. 121 helmets were only mentioned once, 21 had more than 5 mentions, 6 had more than 10 and 1 helmet had more than 50 mentions.

The retail price distribution shown in Fig. 11 was used to ensure the helmets tested were popular and representative of the prices of helmets of cyclists who chose to own or wear a helmet. Once the 13 already selected helmets were included, the survey responses in order of helmet popularity were used to fit the price bands which were not yet at capacity. In instances of equal popularity, helmets were selected with the aim of increasing the diversity of the list of 30 cycling helmets for testing (Table 8).

**Table 8:** Using the distribution of retail prices of cycling helmets from the survey responses to identify how many helmets are required to populate each price band

Price band, X (£)	Price band frequency	Helmets from popular retailer best-selling list	Outstanding
0 < *X* £ 20	3	3	0
20 < *X* £ 30	4	4	0
30 < *X* £ 40	3	1	2
40 < *X* £ 50	4	0	4
50 < *X* £ 60	2	1	1
60 < *X* £ 70	2	2	0
70 < *X* £ 80	2	1	1
80 < *X* £ 90	1	1	0
90 < *X* £ 100	2	0	2
100 < *X* £ 110	0	0	0
110 < *X* £ 120	1	0	1
120 < *X* £ 130	1	0	1
130 < *X* £ 140	1	0	1
140 < *X* £ 150	1	0	1
150 < *X* £ 200	2	0	2
*X* > 200	1	0	1

As such, helmets with distinct features and or technologies were chosen in said instances, for example, foldable helmets and helmets that have LEDs. In the event that there were an insufficient number of helmets within a given price band from the survey responses, the initial helmet long list was used to select the best-selling helmet within that price band. Further information relating to the survey responses and helmet pricing can be found in “Appendix 1”. The final list of 30 helmets selected for testing are shown in Table 4 (Fig. 12).

## Appendix 2

A summary of the kinematics of all tests can be found in this section (Table 9). The distributions of kinematics are summarized below.

**Table 9:** Shows the kinematic results for all repeats and all helmet tests (test ID and impact test type: pXR, pYR, pZR, or nYR are shown), in addition to means and coefficients of variation for the peak linear acceleration (PLA), peak rotational velocity (PRV), peak rotational acceleration (PRA), and the brain injury criterion (BrIC).

Test ID	Impact	PLA (g)	PRV (rad/s)	PRA (rad/s^2^)	BrIC
2023-02-13_HalfordsUrban_nYR_04	nYR	161.9	25.7	7.21	0.4591
2023-02-13_HalfordsUrban_nYR_05	nYR	169.6	25.3	6.94	0.4513
2023-02-13_HalfordsUrban_nYR_06	nYR	170.4	25.6	6.95	0.4579
Mean	nYR	167.3	25.5	7.03	0.4561
Coefficient of Variance	nYR	3%	1%	2%	1%
2023-02-13_HalfordsUrban_pXR_01	pXR	202.2	21.9	6.84	0.3399
2023-02-13_HalfordsUrban_pXR_02	pXR	215.7	21.0	7.00	0.3333
2023-02-13_HalfordsUrban_pXR_03	pXR	198.0	22.2	6.40	0.3460
Mean	pXR	205.3	21.7	6.74	0.3397
Coefficient of Variance	pXR	5%	3%	5%	2%
2023-02-13_HalfordsUrban_pYR_01	pYR	196.8	25.5	9.00	0.4518
2023-02-13_HalfordsUrban_pYR_02	pYR	190.3	25.3	7.75	0.4515
2023-02-13_HalfordsUrban_pYR_03	pYR	173.9	25.3	7.37	0.4485
Mean	pYR	187.0	25.3	8.04	0.4506
Coefficient of Variance	pYR	6%	0%	11%	0%
2023-02-13_HalfordsUrban_pZR_04	pZR	149.2	27.0	8.57	0.6012
2023-02-13_HalfordsUrban_pZR_05	pZR	150.8	27.7	8.82	0.6145
2023-02-13_HalfordsUrban_pZR_06	pZR	153.0	29.0	9.67	0.6439
Mean	pZR	151.0	27.9	9.02	0.6199
Coefficient of Variance	pZR	1%	4%	6%	4%
2023-02-20_HalfordsTrail_nYR_04	nYR	96.2	27.5	4.56	0.4964
2023-02-20_HalfordsTrail_nYR_05	nYR	99.6	27.6	4.58	0.4917
2023-02-20_HalfordsTrail_nYR_06	nYR	93.2	29.9	4.89	0.5368
Mean	nYR	96.3	28.3	4.68	0.5083
Coefficient of Variance	nYR	3%	4%	3%	4%
2023-02-20_HalfordsTrail_pXR_01	pXR	173.6	17.5	4.90	0.2783
2023-02-20_HalfordsTrail_pXR_02	pXR	177.2	19.8	5.82	0.3081
2023-02-20_HalfordsTrail_pXR_03	pXR	165.9	19.6	5.36	0.3033
Mean	pXR	172.2	19.0	5.36	0.2965
Coefficient of Variance	pXR	3%	7%	9%	5%
2023-02-20_HalfordsTrail_pYR_03	pYR	154.2	25.5	6.75	0.4526
2023-02-20_HalfordsTrail_pYR_02	pYR	146.6	25.9	6.62	0.4631
2023-02-20_HalfordsTrail_pYR_01	pYR	153.1	26.5	7.51	0.4765
Mean	pYR	151.3	26.0	6.96	0.4641
Coefficient of Variance	pYR	3%	2%	7%	3%
2023-02-20_HalfordsTrail_pZR_06	pZR	125.3	23.4	6.84	0.5558
2023-02-20_HalfordsTrail_pZR_05	pZR	116.3	22.6	5.02	0.5366
2023-02-20_HalfordsTrail_pZR_04	pZR	156.8	20.6	5.37	0.4746
Mean	pZR	132.8	22.2	5.74	0.5223
Coefficient of Variance	pZR	16%	7%	17%	8%
2023-03-01_DHBR2Road_nYR_04	nYR	99.0	27.6	4.40	0.4947
2023-03-01_DHBR2Road_nYR_05	nYR	101.7	28.5	4.93	0.5097
2023-03-01_DHBR2Road_nYR_06	nYR	97.3	27.1	3.88	0.4794
Mean	nYR	99.3	27.7	4.40	0.4946
Coefficient of Variance	nYR	2%	2%	10%	3%
2023-03-01_DHBR2Road_pXR_01	pXR	176.6	18.9	4.01	0.2923
2023-03-01_DHBR2Road_pXR_02	pXR	182.0	18.3	4.43	0.2816
2023-03-01_DHBR2Road_pXR_03	pXR	185.4	17.2	3.52	0.2629
Mean	pXR	181.3	18.1	3.99	0.2789
Coefficient of Variance	pXR	2%	5%	11%	5%
2023-03-01_DHBR2Road_pYR_02	pYR	177.9	25.8	7.73	0.4585
2023-03-01_DHBR2Road_pYR_01	pYR	169.1	25.2	7.16	0.4454
2023-03-01_DHBR2Road_pYR_03	pYR	186.2	25.2	8.29	0.4489
Mean	pYR	177.7	25.4	7.72	0.4509
Coefficient of Variance	pYR	5%	1%	7%	2%
2023-03-01_DHBR2Road_pZR_06	pZR	176.9	24.7	8.85	0.5430
2023-03-01_DHBR2Road_pZR_05	pZR	158.8	23.6	8.17	0.5491
2023-03-01_DHBR2Road_pZR_04	pZR	156.6	26.7	9.27	0.6253
Mean	pZR	164.1	25.0	8.76	0.5725
Coefficient of Variance	pZR	7%	6%	6%	8%
2023-03-01_METCrossoverActive_nYR_04	nYR	98.4	24.3	3.77	0.4391
2023-03-01_METCrossoverActive_nYR_05	nYR	100.9	26.8	4.15	0.4765
2023-03-01_METCrossoverActive_nYR_06	nYR	106.3	26.8	4.43	0.4750
Mean	nYR	101.9	26.0	4.12	0.4635
Coefficient of Variance	nYR	4%	6%	8%	5%
2023-03-01_METCrossoverActive_pXR_01	pXR	157.3	16.4	2.90	0.2538
2023-03-01_METCrossoverActive_pXR_02	pXR	171.0	18.8	4.14	0.2849
2023-03-01_METCrossoverActive_pXR_03	pXR	154.4	19.2	4.38	0.2909
Mean	pXR	160.9	18.1	3.81	0.2765
Coefficient of Variance	pXR	6%	8%	21%	7%
2023-03-01_METCrossoverActive_pYR_03	pYR	137.0	26.9	6.36	0.4831
2023-03-01_METCrossoverActive_pYR_02	pYR	129.9	27.7	6.04	0.4885
2023-03-01_METCrossoverActive_pYR_01	pYR	129.4	27.9	6.14	0.4935
Mean	pYR	132.1	27.5	6.18	0.4884
Coefficient of Variance	pYR	3%	2%	3%	1%
2023-03-01_METCrossoverActive_pZR_06	pZR	138.6	22.4	6.04	0.5317
2023-03-01_METCrossoverActive_pZR_05	pZR	115.9	26.0	6.87	0.5927
2023-03-01_METCrossoverActive_pZR_04	pZR	136.4	23.1	6.43	0.5465
Mean	pZR	130.3	23.8	6.44	0.5570
Coefficient of Variance	pZR	10%	8%	6%	6%
2023-03-01_METIdolo_nYR_04	nYR	103.7	27.7	4.32	0.4967
2023-03-01_METIdolo_nYR_05	nYR	96.1	26.6	3.66	0.4755
2023-03-01_METIdolo_nYR_06	nYR	97.8	27.3	4.36	0.4863
Mean	nYR	99.2	27.2	4.11	0.4862
Coefficient of Variance	nYR	4%	2%	10%	2%
2023-03-01_METIdolo_pXR_01	pXR	166.4	19.2	4.29	0.2919
2023-03-01_METIdolo_pXR_02	pXR	162.1	18.5	4.28	0.2823
2023-03-01_METIdolo_pXR_03	pXR	157.6	18.3	4.07	0.2811
Mean	pXR	162.0	18.7	4.21	0.2851
Coefficient of Variance	pXR	3%	3%	3%	2%
2023-03-01_METIdolo_pYR_03	pYR	135.6	26.6	5.85	0.4773
2023-03-01_METIdolo_pYR_02	pYR	129.0	26.5	6.16	0.4782
2023-03-01_METIdolo_pYR_01	pYR	127.5	26.9	5.78	0.4773
Mean	pYR	130.7	26.7	5.93	0.4776
Coefficient of Variance	pYR	3%	1%	3%	0%
2023-03-01_METIdolo_pZR_06	pZR	132.0	23.5	6.62	0.5634
2023-03-01_METIdolo_pZR_05	pZR	145.0	23.5	6.82	0.5347
2023-03-01_METIdolo_pZR_04	pZR	127.6	23.8	6.63	0.5745
Mean	pZR	134.9	23.6	6.69	0.5575
Coefficient of Variance	pZR	7%	1%	2%	4%
2023-03-08_HalfordsSport_nYR_04	nYR	102.0	22.0	3.51	0.3932
2023-03-08_HalfordsSport_nYR_05	nYR	97.2	21.0	3.24	0.3766
2023-03-08_HalfordsSport_nYR_06	nYR	107.2	23.2	3.66	0.4131
Mean	nYR	102.2	22.1	3.47	0.3943
Coefficient of Variance	nYR	5%	5%	6%	5%
2023-03-08_HalfordsSport_pXR_01	pXR	154.9	19.1	4.73	0.2991
2023-03-08_HalfordsSport_pXR_02	pXR	159.2	17.9	4.60	0.2847
2023-03-08_HalfordsSport_pXR_03	pXR	177.5	18.7	4.70	0.2934
Mean	pXR	163.9	18.6	4.68	0.2924
Coefficient of Variance	pXR	7%	3%	2%	2%
2023-03-08_HalfordsSport_pYR_03	pYR	135.8	26.2	6.23	0.4690
2023-03-08_HalfordsSport_pYR_02	pYR	136.8	26.4	6.43	0.4680
2023-03-08_HalfordsSport_pYR_01	pYR	134.0	26.0	5.78	0.4610
Mean	pYR	135.5	26.2	6.15	0.4660
Coefficient of Variance	pYR	1%	1%	5%	1%
2023-03-08_HalfordsSport_pZR_06	pZR	120.6	24.0	5.74	0.5664
2023-03-08_HalfordsSport_pZR_05	pZR	124.9	23.4	5.16	0.5622
2023-03-08_HalfordsSport_pZR_04	pZR	119.4	24.2	5.60	0.5755
Mean	pZR	121.6	23.9	5.50	0.5680
Coefficient of Variance	pZR	2%	2%	6%	1%
2023-03-09_BTwin500_nYR_04	nYR	199.5	24.4	8.46	0.4411
2023-03-09_BTwin500_nYR_05	nYR	197.1	24.3	8.21	0.4372
2023-03-09_BTwin500_nYR_06	nYR	189.0	25.0	6.45	0.4505
Mean	nYR	195.2	24.6	7.70	0.4429
Coefficient of Variance	nYR	3%	1%	14%	2%
2023-03-09_BTwin500_pXR_01	pXR	184.0	20.6	5.72	0.3285
2023-03-09_BTwin500_pXR_02	pXR	172.2	19.1	4.50	0.2986
2023-03-09_BTwin500_pXR_03	pXR	184.9	21.3	6.03	0.3303
Mean	pXR	180.4	20.3	5.42	0.3192
Coefficient of Variance	pXR	4%	6%	15%	6%
2023-03-09_BTwin500_pYR_03	pYR	177.0	25.1	6.79	0.4479
2023-03-09_BTwin500_pYR_02	pYR	163.3	25.6	7.37	0.4527
2023-03-09_BTwin500_pYR_01	pYR	168.8	25.5	7.14	0.4517
Mean	pYR	169.7	25.4	7.10	0.4508
Coefficient of Variance	pYR	4%	1%	4%	1%
2023-03-09_BTwin500_pZR_06	pZR	156.1	20.3	6.40	0.5002
2023-03-09_BTwin500_pZR_05	pZR	132.1	20.0	4.36	0.4818
2023-03-09_BTwin500_pZR_04	pZR	129.1	20.8	4.40	0.5159
Mean	pZR	139.1	20.4	5.05	0.4993
Coefficient of Variance	pZR	11%	2%	23%	3%
2023-03-09_HalfordsEssentials_nYR_04	nYR	120.8	27.1	4.74	0.4815
2023-03-09_HalfordsEssentials_nYR_05	nYR	145.9	24.5	5.39	0.4403
2023-03-09_HalfordsEssentials_nYR_06	nYR	121.5	26.5	4.98	0.4762
Mean	nYR	129.4	26.0	5.04	0.4660
Coefficient of Variance	nYR	11%	5%	7%	5%
2023-03-09_HalfordsEssentials_pXR_01	pXR	194.6	18.5	4.90	0.2905
2023-03-09_HalfordsEssentials_pXR_02	pXR	184.0	18.6	4.58	0.2867
2023-03-09_HalfordsEssentials_pXR_03	pXR	193.7	16.5	4.58	0.2612
Mean	pXR	190.8	17.9	4.69	0.2794
Coefficient of Variance	pXR	3%	7%	4%	6%
2023-03-09_HalfordsEssentials_pYR_03	pYR	168.4	26.1	7.84	0.4640
2023-03-09_HalfordsEssentials_pYR_02	pYR	214.5	26.5	9.77	0.4659
2023-03-09_HalfordsEssentials_pYR_01	pYR	179.1	27.2	8.71	0.4798
Mean	pYR	187.3	26.6	8.77	0.4699
Coefficient of Variance	pYR	13%	2%	11%	2%
2023-03-09_HalfordsEssentials_pZR_04	pZR	143.4	21.0	6.50	0.4734
2023-03-09_HalfordsEssentials_pZR_05	pZR	156.8	20.4	6.44	0.4692
2023-03-09_HalfordsEssentials_pZR_06	pZR	159.9	22.0	6.89	0.5076
Mean	pZR	153.4	21.1	6.61	0.4834
Coefficient of Variance	pZR	6%	4%	4%	4%
2023-03-09_SpecializedEchelonIIMIPS_nYR_04	nYR	100.6	12.3	1.66	0.2217
2023-03-09_SpecializedEchelonIIMIPS_nYR_05	nYR	105.4	12.2	1.59	0.2233
2023-03-09_SpecializedEchelonIIMIPS_nYR_06	nYR	95.3	13.3	1.49	0.2373
Mean	nYR	100.4	12.6	1.58	0.2274
Coefficient of Variance	nYR	5%	5%	5%	4%
2023-03-09_SpecializedEchelonIIMIPS_pXR_01	pXR	175.8	8.6	3.54	0.1889
2023-03-09_SpecializedEchelonIIMIPS_pXR_02	pXR	169.4	7.6	2.66	0.1485
2023-03-09_SpecializedEchelonIIMIPS_pXR_03	pXR	154.1	9.2	2.94	0.1846
Mean	pXR	166.4	8.5	3.05	0.1740
Coefficient of Variance	pXR	7%	9%	15%	13%
2023-03-09_SpecializedEchelonIIMIPS_pYR_03	pYR	135.3	14.9	2.62	0.2717
2023-03-09_SpecializedEchelonIIMIPS_pYR_02	pYR	140.2	14.1	2.56	0.2564
2023-03-09_SpecializedEchelonIIMIPS_pYR_01	pYR	137.1	18.0	4.15	0.3265
Mean	pYR	137.5	15.7	3.11	0.2848
Coefficient of Variance	pYR	2%	13%	29%	13%
2023-03-09_SpecializedEchelonIIMIPS_pZR_06	pZR	126.7	16.1	4.33	0.3307
2023-03-09_SpecializedEchelonIIMIPS_pZR_05	pZR	113.6	15.6	4.95	0.3037
2023-03-09_SpecializedEchelonIIMIPS_pZR_04	pZR	126.4	16.2	4.44	0.3487
Mean	pZR	122.2	16.0	4.57	0.3277
Coefficient of Variance	pZR	6%	2%	7%	7%
2023-04-03_LazerCompact_nYR_04	nYR	174.0	28.3	7.34	0.5071
2023-04-03_LazerCompact_nYR_05	nYR	169.8	28.0	7.22	0.5009
2023-04-03_LazerCompact_nYR_06	nYR	160.7	26.9	6.32	0.4805
Mean	nYR	168.2	27.7	6.96	0.4962
Coefficient of Variance	nYR	4%	3%	8%	3%
2023-04-03_LazerCompact_pXR_01	pXR	206.7	20.1	4.57	0.3087
2023-04-03_LazerCompact_pXR_02	pXR	212.2	21.0	5.41	0.3193
2023-04-03_LazerCompact_pXR_03	pXR	220.9	19.6	4.61	0.3024
Mean	pXR	213.3	20.2	4.86	0.3101
Coefficient of Variance	pXR	3%	4%	10%	3%
2023-04-03_LazerCompact_pYR_03	pYR	153.4	28.7	8.92	0.4950
2023-04-03_LazerCompact_pYR_02	pYR	154.8	28.3	9.23	0.5027
2023-04-03_LazerCompact_pYR_01	pYR	149.3	26.5	7.67	0.4719
Mean	pYR	152.5	27.8	8.60	0.4899
Coefficient of Variance	pYR	2%	4%	10%	3%
2023-04-03_LazerCompact_pZR_06	pZR	168.6	24.3	8.36	0.5631
2023-04-03_LazerCompact_pZR_05	pZR	154.8	23.8	7.43	0.5570
2023-04-03_LazerCompact_pZR_04	pZR	160.2	26.3	7.83	0.6124
Mean	pZR	161.2	24.8	7.87	0.5775
Coefficient of Variance	pZR	4%	5%	6%	5%
2023-04-04_MavicAksium_nYR_04	nYR	89.2	25.8	3.16	0.4675
2023-04-04_MavicAksium_nYR_05	nYR	97.4	24.6	2.96	0.4386
2023-04-04_MavicAksium_nYR_06	nYR	108.1	24.5	3.74	0.4412
Mean	nYR	98.2	25.0	3.29	0.4491
Coefficient of Variance	nYR	10%	3%	12%	4%
2023-04-04_MavicAksium_pXR_01	pXR	133.7	21.2	4.39	0.3289
2023-04-04_MavicAksium_pXR_02	pXR	132.0	21.0	4.52	0.3211
2023-04-04_MavicAksium_pXR_03	pXR	136.1	17.2	3.36	0.2804
Mean	pXR	133.9	19.8	4.09	0.3102
Coefficient of Variance	pXR	2%	11%	16%	8%
2023-04-04_MavicAksium_pYR_03	pYR	135.0	22.6	4.75	0.4031
2023-04-04_MavicAksium_pYR_02	pYR	140.6	23.0	5.03	0.4055
2023-04-04_MavicAksium_pYR_01	pYR	142.7	23.1	4.97	0.4067
Mean	pYR	139.4	22.9	4.92	0.4051
Coefficient of Variance	pYR	2%	1%	2%	0%
2023-04-04_MavicAksium_pZR_06	pZR	127.4	23.0	6.12	0.5396
2023-04-04_MavicAksium_pZR_05	pZR	127.0	25.1	6.19	0.5761
2023-04-04_MavicAksium_pZR_04	pZR	121.1	24.1	6.05	0.5640
Mean	pZR	125.2	24.0	6.12	0.5599
Coefficient of Variance	pZR	3%	4%	1%	3%
2023-04-26_GiroAngon_nYR_04	nYR	146.1	28.9	6.36	0.5127
2023-04-26_GiroAngon_nYR_05	nYR	145.6	28.3	5.68	0.5012
2023-04-26_GiroAngon_nYR_06	nYR	140.7	27.5	5.48	0.4931
Mean	nYR	144.1	28.2	5.84	0.5023
Coefficient of Variance	nYR	2%	2%	8%	2%
2023-04-26_GiroAngon_pXR_01	pXR	180.8	19.1	4.29	0.2955
2023-04-26_GiroAngon_pXR_02	pXR	178.0	19.2	4.07	0.2960
2023-04-26_GiroAngon_pXR_03	pXR	176.7	19.1	3.91	0.2917
Mean	pXR	178.5	19.1	4.09	0.2944
Coefficient of Variance	pXR	1%	0%	5%	1%
2023-04-26_GiroAngon_pYR_03	pYR	155.9	23.5	6.21	0.4194
2023-04-26_GiroAngon_pYR_02	pYR	141.2	23.6	6.68	0.4209
2023-04-26_GiroAngon_pYR_01	pYR	146.9	23.8	7.07	0.4209
Mean	pYR	148.0	23.6	6.65	0.4204
Coefficient of Variance	pYR	5%	1%	7%	0%
2023-04-26_GiroAngon_pZR_06	pZR	158.8	28.4	9.85	0.6529
2023-04-26_GiroAngon_pZR_05	pZR	156.4	28.0	9.24	0.6446
2023-04-26_GiroAngon_pZR_04	pZR	154.2	26.1	9.14	0.6404
Mean	pZR	156.5	27.5	9.41	0.6460
Coefficient of Variance	pZR	1%	4%	4%	1%
2023-04-27_GiroAngonMIPS_nYR_04	nYR	137.7	23.4	4.17	0.4163
2023-04-27_GiroAngonMIPS_nYR_05	nYR	143.5	24.3	4.42	0.4352
2023-04-27_GiroAngonMIPS_nYR_06	nYR	145.3	26.0	5.56	0.4675
Mean	nYR	142.2	24.6	4.72	0.4397
Coefficient of Variance	nYR	3%	6%	16%	6%
2023-04-27_GiroAngonMIPS_pXR_01	pXR	162.3	14.4	3.89	0.2274
2023-04-27_GiroAngonMIPS_pXR_02	pXR	170.9	13.7	3.95	0.2093
2023-04-27_GiroAngonMIPS_pXR_03	pXR	162.7	16.7	4.05	0.2584
Mean	pXR	165.3	14.9	3.96	0.2317
Coefficient of Variance	pXR	3%	10%	2%	11%
2023-04-27_GiroAngonMIPS_pYR_03	pYR	148.2	24.9	5.51	0.4388
2023-04-27_GiroAngonMIPS_pYR_02	pYR	155.3	25.1	5.84	0.4456
2023-04-27_GiroAngonMIPS_pYR_01	pYR	149.3	25.1	6.12	0.4447
Mean	pYR	150.9	25.0	5.82	0.4430
Coefficient of Variance	pYR	3%	0%	5%	1%
2023-04-27_GiroAngonMIPS_pZR_06	pZR	135.8	22.6	5.60	0.5268
2023-04-27_GiroAngonMIPS_pZR_05	pZR	133.1	21.4	5.06	0.4967
2023-04-27_GiroAngonMIPS_pZR_04	pZR	140.3	22.3	5.90	0.5175
Mean	pZR	136.4	22.1	5.52	0.5137
Coefficient of Variance	pZR	3%	3%	8%	3%
2023-04-27_LazerTonicMIPS_nYR_04	nYR	114.1	24.8	3.82	0.4443
2023-04-27_LazerTonicMIPS_nYR_05	nYR	103.0	23.1	2.92	0.4107
2023-04-27_LazerTonicMIPS_nYR_06	nYR	104.5	22.6	3.05	0.4049
Mean	nYR	107.2	23.5	3.26	0.4200
Coefficient of Variance	nYR	6%	5%	15%	5%
2023-04-27_LazerTonicMIPS_pXR_01	pXR	138.4	13.1	2.44	0.2222
2023-04-27_LazerTonicMIPS_pXR_02	pXR	144.4	14.2	2.72	0.2242
2023-04-27_LazerTonicMIPS_pXR_03	pXR	133.6	14.5	2.50	0.2359
Mean	pXR	138.8	13.9	2.55	0.2274
Coefficient of Variance	pXR	4%	5%	6%	3%
2023-04-27_LazerTonicMIPS_pYR_03	pYR	111.7	20.7	3.54	0.3725
2023-04-27_LazerTonicMIPS_pYR_02	pYR	116.0	20.0	3.48	0.3574
2023-04-27_LazerTonicMIPS_pYR_01	pYR	117.6	23.7	3.91	0.4252
Mean	pYR	115.1	21.5	3.64	0.3850
Coefficient of Variance	pYR	3%	9%	6%	9%
2023-04-27_LazerTonicMIPS_pZR_06	pZR	126.1	18.2	4.27	0.4309
2023-04-27_LazerTonicMIPS_pZR_05	pZR	122.3	16.5	3.43	0.3802
2023-04-27_LazerTonicMIPS_pZR_04	pZR	120.5	19.6	4.18	0.4348
Mean	pZR	123.0	18.1	3.96	0.4153
Coefficient of Variance	pZR	2%	9%	12%	7%
2023-04-28_LazerTonic_nYR_04	nYR	95.8	26.4	4.16	0.4740
2023-04-28_LazerTonic_nYR_05	nYR	95.1	25.7	3.94	0.4641
2023-04-28_LazerTonic_nYR_06	nYR	99.0	26.8	4.13	0.4795
Mean	nYR	96.6	26.3	4.08	0.4725
Coefficient of Variance	nYR	2%	2%	3%	2%
2023-04-28_LazerTonic_pXR_01	pXR	137.1	19.0	3.25	0.2946
2023-04-28_LazerTonic_pXR_02	pXR	144.1	19.9	3.47	0.3069
2023-04-28_LazerTonic_pXR_03	pXR	141.5	19.6	3.22	0.3042
Mean	pXR	140.9	19.5	3.31	0.3019
Coefficient of Variance	pXR	3%	2%	4%	2%
2023-04-28_LazerTonic_pYR_03	pYR	127.3	25.8	5.52	0.4600
2023-04-28_LazerTonic_pYR_02	pYR	136.9	25.4	6.03	0.4576
2023-04-28_LazerTonic_pYR_01	pYR	123.1	26.3	5.82	0.4625
Mean	pYR	129.1	25.9	5.79	0.4601
Coefficient of Variance	pYR	5%	2%	4%	1%
2023-04-28_LazerTonic_pZR_06	pZR	135.7	24.9	7.25	0.5731
2023-04-28_LazerTonic_pZR_05	pZR	132.7	25.2	6.58	0.5819
2023-04-28_LazerTonic_pZR_04	pZR	128.1	24.7	6.61	0.5729
Mean	pZR	132.2	24.9	6.81	0.5760
Coefficient of Variance	pZR	3%	1%	6%	1%
2023-05-02_BellFormulaLEDMIPS_nYR_04	nYR	92.3	23.9	2.73	0.4308
2023-05-02_BellFormulaLEDMIPS_nYR_05	nYR	110.2	25.5	3.89	0.4537
2023-05-02_BellFormulaLEDMIPS_nYR_06	nYR	159.7	24.1	5.26	0.4279
Mean	nYR	120.7	24.5	3.96	0.4375
Coefficient of Variance	nYR	29%	4%	32%	3%
2023-05-02_BellFormulaLEDMIPS_pXR_01	pXR	137.0	15.0	3.12	0.2350
2023-05-02_BellFormulaLEDMIPS_pXR_02	pXR	146.2	21.3	4.40	0.3252
2023-05-02_BellFormulaLEDMIPS_pXR_03	pXR	130.8	14.8	3.35	0.2262
Mean	pXR	138.0	17.0	3.62	0.2621
Coefficient of Variance	pXR	6%	22%	19%	21%
2023-05-02_BellFormulaLEDMIPS_pYR_03	pYR	143.6	25.0	5.71	0.4472
2023-05-02_BellFormulaLEDMIPS_pYR_02	pYR	129.2	24.5	5.04	0.4458
2023-05-02_BellFormulaLEDMIPS_pYR_01	pYR	129.8	23.0	4.55	0.4162
Mean	pYR	134.2	24.2	5.10	0.4364
Coefficient of Variance	pYR	6%	4%	11%	4%
2023-05-02_BellFormulaLEDMIPS_pZR_06	pZR	110.4	26.6	5.02	0.6165
2023-05-02_BellFormulaLEDMIPS_pZR_05	pZR	107.8	26.2	5.74	0.6028
2023-05-02_BellFormulaLEDMIPS_pZR_04	pZR	121.1	20.4	5.06	0.4629
Mean	pZR	113.1	24.4	5.27	0.5607
Coefficient of Variance	pZR	6%	14%	8%	15%
2023-05-10_VanRyselRoadR900_nYR_04	nYR	154.6	28.1	6.42	0.5018
2023-05-10_VanRyselRoadR900_nYR_05	nYR	151.0	28.1	5.75	0.5006
2023-05-10_VanRyselRoadR900_nYR_06	nYR	151.3	28.4	5.92	0.5056
Mean	nYR	152.3	28.2	6.03	0.5027
Coefficient of Variance	nYR	1%	1%	6%	1%
2023-05-10_VanRyselRoadR900_pXR_01	pXR	167.7	19.3	4.15	0.2968
2023-05-10_VanRyselRoadR900_pXR_02	pXR	177.2	18.8	3.46	0.2883
2023-05-10_VanRyselRoadR900_pXR_03	pXR	177.1	20.2	3.94	0.3094
Mean	pXR	174.0	19.4	3.85	0.2982
Coefficient of Variance	pXR	3%	4%	9%	4%
2023-05-10_VanRyselRoadR900_pYR_03	pYR	173.6	27.5	8.45	0.4863
2023-05-10_VanRyselRoadR900_pYR_02	pYR	175.8	28.4	9.89	0.5034
2023-05-10_VanRyselRoadR900_pYR_01	pYR	179.2	27.8	8.86	0.4953
Mean	pYR	176.2	27.9	9.07	0.4950
Coefficient of Variance	pYR	2%	2%	8%	2%
2023-05-10_VanRyselRoadR900_pZR_06	pZR	143.9	26.7	5.95	0.5986
2023-05-10_VanRyselRoadR900_pZR_05	pZR	131.2	25.6	6.07	0.5764
2023-05-10_VanRyselRoadR900_pZR_04	pZR	137.6	25.2	5.82	0.5760
Mean	pZR	137.6	25.8	5.95	0.5837
Coefficient of Variance	pZR	5%	3%	2%	2%
2023-05-18_ABUSVillite2Ace_nYR_04	nYR	160.4	24.3	5.03	0.4333
2023-05-18_ABUSVillite2Ace_nYR_05	nYR	162.3	24.1	4.86	0.4317
2023-05-18_ABUSVillite2Ace_nYR_06	nYR	161.2	25.1	5.88	0.4506
Mean	nYR	161.3	24.5	5.25	0.4385
Coefficient of Variance	nYR	1%	2%	10%	2%
2023-05-18_ABUSVillite2Ace_pXR_01	pXR	163.4	14.9	3.45	0.2352
2023-05-18_ABUSVillite2Ace_pXR_02	pXR	161.1	15.2	3.66	0.2412
2023-05-18_ABUSVillite2Ace_pXR_03	pXR	162.9	15.6	3.69	0.2395
Mean	pXR	162.5	15.3	3.60	0.2386
Coefficient of Variance	pXR	1%	2%	4%	1%
2023-05-18_ABUSVillite2Ace_pYR_03	pYR	155.1	22.8	5.48	0.4027
2023-05-18_ABUSVillite2Ace_pYR_02	pYR	143.5	23.0	5.16	0.4064
2023-05-18_ABUSVillite2Ace_pYR_01	pYR	139.7	23.4	4.82	0.4132
Mean	pYR	146.1	23.0	5.15	0.4074
Coefficient of Variance	pYR	6%	1%	6%	1%
2023-05-18_ABUSVillite2Ace_pZR_06	pZR	142.4	25.8	7.13	0.5981
2023-05-18_ABUSVillite2Ace_pZR_05	pZR	144.4	23.1	6.32	0.5359
2023-05-18_ABUSVillite2Ace_pZR_04	pZR	141.4	24.8	5.35	0.5763
Mean	pZR	142.7	24.6	6.27	0.5701
Coefficient of Variance	pZR	1%	6%	14%	6%
2023-05-18_GiroAgilisMIPS_nYR_04	nYR	148.5	25.3	6.30	0.4491
2023-05-18_GiroAgilisMIPS_nYR_05	nYR	145.3	26.0	6.63	0.4586
2023-05-18_GiroAgilisMIPS_nYR_06	nYR	144.5	25.2	6.35	0.4517
Mean	nYR	146.1	25.5	6.42	0.4531
Coefficient of Variance	nYR	1%	2%	3%	1%
2023-05-18_GiroAgilisMIPS_pXR_01	pXR	130.5	17.7	5.01	0.2674
2023-05-18_GiroAgilisMIPS_pXR_02	pXR	122.2	11.9	3.94	0.1824
2023-05-18_GiroAgilisMIPS_pXR_03	pXR	115.9	15.5	4.62	0.2368
Mean	pXR	122.9	15.0	4.52	0.2288
Coefficient of Variance	pXR	6%	19%	12%	19%
2023-05-18_GiroAgilisMIPS_pYR_03	pYR	149.0	25.4	5.51	0.4486
2023-05-18_GiroAgilisMIPS_pYR_02	pYR	148.4	21.1	4.86	0.3763
2023-05-18_GiroAgilisMIPS_pYR_01	pYR	147.2	23.2	5.15	0.4092
Mean	pYR	148.2	23.2	5.18	0.4114
Coefficient of Variance	pYR	1%	9%	6%	9%
2023-05-18_GiroAgilisMIPS_pZR_06	pZR	134.9	19.8	5.59	0.4446
2023-05-18_GiroAgilisMIPS_pZR_05	pZR	126.3	19.7	4.82	0.4314
2023-05-18_GiroAgilisMIPS_pZR_04	pZR	117.3	20.7	4.71	0.4534
Mean	pZR	126.2	20.1	5.04	0.4431
Coefficient of Variance	pZR	7%	3%	10%	2%
2023-05-19_OveradePlixi_nYR_04	nYR	154.2	16.8	4.85	0.3090
2023-05-19_OveradePlixi_nYR_05	nYR	152.5	17.4	4.94	0.3154
2023-05-19_OveradePlixi_nYR_06	nYR	148.5	18.0	5.09	0.3324
Mean	nYR	151.7	17.4	4.96	0.3189
Coefficient of Variance	nYR	2%	3%	2%	4%
2023-05-19_OveradePlixi_pXR_01	pXR	188.8	18.9	5.92	0.3051
2023-05-19_OveradePlixi_pXR_02	pXR	193.2	19.5	6.19	0.3089
2023-05-19_OveradePlixi_pXR_03	pXR	188.6	19.7	5.23	0.3093
Mean	pXR	190.2	19.4	5.78	0.3078
Coefficient of Variance	pXR	1%	2%	9%	1%
2023-05-19_OveradePlixi_pYR_03	pYR	217.6	24.2	8.78	0.4342
2023-05-19_OveradePlixi_pYR_02	pYR	204.4	24.5	7.55	0.4377
2023-05-19_OveradePlixi_pYR_01	pYR	213.5	24.2	9.08	0.4321
Mean	pYR	211.8	24.3	8.47	0.4347
Coefficient of Variance	pYR	3%	1%	10%	1%
2023-05-19_OveradePlixi_pZR_06	pZR	163.1	28.2	9.73	0.6419
2023-05-19_OveradePlixi_pZR_05	pZR	143.5	25.1	8.09	0.5844
2023-05-19_OveradePlixi_pZR_04	pZR	153.9	26.7	8.75	0.6216
Mean	pZR	153.5	26.7	8.85	0.6160
Coefficient of Variance	pZR	6%	6%	9%	5%
2023-05-23_BontragerVelocisMIPS_nYR_04	nYR	142.5	25.1	5.03	0.4474
2023-05-23_BontragerVelocisMIPS_nYR_05	nYR	133.0	25.5	5.09	0.4554
2023-05-23_BontragerVelocisMIPS_nYR_06	nYR	129.3	23.2	4.33	0.4178
Mean	nYR	134.9	24.6	4.82	0.4402
Coefficient of Variance	nYR	4%	4%	7%	4%
2023-05-23_BontragerVelocisMIPS_pXR_01	pXR	130.7	14.9	3.26	0.2316
2023-05-23_BontragerVelocisMIPS_pXR_02	pXR	135.6	10.4	2.70	0.1634
2023-05-23_BontragerVelocisMIPS_pXR_03	pXR	130.1	12.5	2.84	0.1956
Mean	pXR	132.1	12.6	2.93	0.1969
Coefficient of Variance	pXR	2%	18%	10%	17%
2023-05-23_BontragerVelocisMIPS_pYR_03	pYR	122.8	24.9	4.33	0.4445
2023-05-23_BontragerVelocisMIPS_pYR_02	pYR	115.7	24.2	4.37	0.4346
2023-05-23_BontragerVelocisMIPS_pYR_01	pYR	113.2	21.7	3.49	0.3837
Mean	pYR	117.2	23.6	4.07	0.4209
Coefficient of Variance	pYR	4%	7%	12%	8%
2023-05-23_BontragerVelocisMIPS_pZR_06	pZR	95.5	19.9	3.21	0.4249
2023-05-23_BontragerVelocisMIPS_pZR_05	pZR	109.0	17.8	3.86	0.4062
2023-05-23_BontragerVelocisMIPS_pZR_04	pZR	100.7	17.6	3.29	0.3751
Mean	pZR	101.7	18.4	3.45	0.4021
Coefficient of Variance	pZR	7%	7%	10%	6%
2023-05-23_KaskProtone_nYR_04	nYR	164.8	25.0	6.13	0.4470
2023-05-23_KaskProtone_nYR_05	nYR	165.1	24.6	6.54	0.4382
2023-05-23_KaskProtone_nYR_06	nYR	166.6	25.1	6.15	0.4478
Mean	nYR	165.5	24.9	6.27	0.4444
Coefficient of Variance	nYR	1%	1%	4%	1%
2023-05-23_KaskProtone_pXR_01	pXR	157.3	21.4	5.15	0.3366
2023-05-23_KaskProtone_pXR_02	pXR	156.5	20.6	4.85	0.3274
2023-05-23_KaskProtone_pXR_03	pXR	153.5	21.1	4.92	0.3317
Mean	pXR	155.8	21.0	4.97	0.3319
Coefficient of Variance	pXR	1%	2%	3%	1%
2023-05-23_KaskProtone_pYR_03	pYR	170.5	25.3	6.44	0.4501
2023-05-23_KaskProtone_pYR_02	pYR	169.9	25.4	6.54	0.4505
2023-05-23_KaskProtone_pYR_01	pYR	157.6	25.0	6.17	0.4447
Mean	pYR	166.0	25.2	6.38	0.4484
Coefficient of Variance	pYR	4%	1%	3%	1%
2023-05-23_KaskProtone_pZR_06	pZR	122.3	25.6	6.57	0.5906
2023-05-23_KaskProtone_pZR_05	pZR	141.9	30.2	8.00	0.6845
2023-05-23_KaskProtone_pZR_04	pZR	132.0	26.8	5.66	0.6219
Mean	pZR	132.0	27.5	6.74	0.6323
Coefficient of Variance	pZR	7%	9%	18%	8%
2023-05-23_LazerCompactDLXMIPS_nYR_04	nYR	154.9	25.0	5.50	0.4475
2023-05-23_LazerCompactDLXMIPS_nYR_05	nYR	175.2	24.8	5.40	0.4404
2023-05-23_LazerCompactDLXMIPS_nYR_06	nYR	159.4	25.1	4.87	0.4410
Mean	nYR	163.2	25.0	5.26	0.4430
Coefficient of Variance	nYR	7%	1%	6%	1%
2023-05-23_LazerCompactDLXMIPS_pXR_01	pXR	182.3	9.0	2.78	0.1704
2023-05-23_LazerCompactDLXMIPS_pXR_02	pXR	184.4	9.9	2.74	0.1829
2023-05-23_LazerCompactDLXMIPS_pXR_03	pXR	178.1	10.1	2.85	0.1847
Mean	pXR	181.6	9.7	2.79	0.1793
Coefficient of Variance	pXR	2%	6%	2%	4%
2023-05-23_LazerCompactDLXMIPS_pYR_03	pYR	143.4	23.6	6.26	0.4197
2023-05-23_LazerCompactDLXMIPS_pYR_02	pYR	135.5	24.1	5.43	0.4136
2023-05-23_LazerCompactDLXMIPS_pYR_01	pYR	139.0	22.9	5.26	0.4153
Mean	pYR	139.3	23.5	5.65	0.4162
Coefficient of Variance	pYR	3%	3%	9%	1%
2023-05-23_LazerCompactDLXMIPS_pZR_06	pZR	175.6	18.3	7.45	0.4156
2023-05-23_LazerCompactDLXMIPS_pZR_05	pZR	159.1	18.5	6.54	0.4259
2023-05-23_LazerCompactDLXMIPS_pZR_04	pZR	159.5	16.7	6.04	0.3794
Mean	pZR	164.7	17.8	6.68	0.4069
Coefficient of Variance	pZR	6%	5%	11%	6%
2023-05-24_BontragerSolstice_nYR_04	nYR	143.8	24.7	5.02	0.4566
2023-05-24_BontragerSolstice_nYR_05	nYR	151.5	24.9	5.08	0.4450
2023-05-24_BontragerSolstice_nYR_06	nYR	140.9	24.8	4.56	0.4482
Mean	nYR	145.4	24.8	4.89	0.4499
Coefficient of Variance	nYR	4%	0%	6%	1%
2023-05-24_BontragerSolstice_pXR_01	pXR	172.4	21.2	4.39	0.3204
2023-05-24_BontragerSolstice_pXR_02	pXR	156.6	20.5	4.75	0.3085
2023-05-24_BontragerSolstice_pXR_03	pXR	170.0	20.7	5.37	0.3134
Mean	pXR	166.3	20.8	4.83	0.3141
Coefficient of Variance	pXR	5%	2%	10%	2%
2023-05-24_BontragerSolstice_pYR_03	pYR	167.9	26.7	7.69	0.4751
2023-05-24_BontragerSolstice_pYR_02	pYR	154.6	27.2	7.77	0.4843
2023-05-24_BontragerSolstice_pYR_01	pYR	158.8	27.0	7.78	0.4793
Mean	pYR	160.4	26.9	7.75	0.4796
Coefficient of Variance	pYR	4%	1%	1%	1%
2023-05-24_BontragerSolstice_pZR_06	pZR	121.0	18.7	5.69	0.4451
2023-05-24_BontragerSolstice_pZR_05	pZR	108.1	19.2	7.67	0.4737
2023-05-24_BontragerSolstice_pZR_04	pZR	97.6	20.7	6.81	0.5052
Mean	pZR	108.9	19.5	6.72	0.4747
Coefficient of Variance	pZR	11%	5%	15%	6%
2023-05-24_GiroSyntheMIPS_nYR_04	nYR	132.3	24.6	4.39	0.4428
2023-05-24_GiroSyntheMIPS_nYR_05	nYR	130.3	24.9	4.83	0.4469
2023-05-24_GiroSyntheMIPS_nYR_06	nYR	130.4	24.0	4.42	0.4300
Mean	nYR	131.0	24.5	4.55	0.4399
Coefficient of Variance	nYR	1%	2%	5%	2%
2023-05-24_GiroSyntheMIPS_pXR_01	pXR	115.7	17.4	3.62	0.2740
2023-05-24_GiroSyntheMIPS_pXR_02	pXR	125.4	16.5	3.33	0.2586
2023-05-24_GiroSyntheMIPS_pXR_03	pXR	120.1	18.8	4.17	0.2909
Mean	pXR	120.4	17.5	3.71	0.2745
Coefficient of Variance	pXR	4%	7%	12%	6%
2023-05-24_GiroSyntheMIPS_pYR_03	pYR	117.4	24.4	4.79	0.4328
2023-05-24_GiroSyntheMIPS_pYR_02	pYR	127.1	24.2	4.95	0.4258
2023-05-24_GiroSyntheMIPS_pYR_01	pYR	125.0	23.4	4.51	0.4145
Mean	pYR	123.1	24.0	4.75	0.4244
Coefficient of Variance	pYR	4%	2%	5%	2%
2023-05-24_GiroSyntheMIPS_pZR_06	pZR	103.5	26.4	5.52	0.6006
2023-05-24_GiroSyntheMIPS_pZR_05	pZR	109.6	23.1	4.22	0.5258
2023-05-24_GiroSyntheMIPS_pZR_04	pZR	108.7	25.0	4.72	0.5731
Mean	pZR	107.3	24.8	4.82	0.5665
Coefficient of Variance	pZR	3%	7%	14%	7%
2023-05-24_KaskMojito3_nYR_04	nYR	166.0	24.9	5.20	0.4523
2023-05-24_KaskMojito3_nYR_05	nYR	157.3	25.5	5.66	0.4554
2023-05-24_KaskMojito3_nYR_06	nYR	163.2	26.2	5.24	0.4677
Mean	nYR	162.2	25.6	5.36	0.4585
Coefficient of Variance	nYR	3%	3%	5%	2%
2023-05-24_KaskMojito3_pXR_01	pXR	152.7	22.1	5.07	0.3456
2023-05-24_KaskMojito3_pXR_02	pXR	153.1	22.4	5.45	0.3456
2023-05-24_KaskMojito3_pXR_03	pXR	155.7	23.3	5.48	0.3568
Mean	pXR	153.8	22.6	5.33	0.3493
Coefficient of Variance	pXR	1%	3%	4%	2%
2023-05-24_KaskMojito3_pYR_03	pYR	129.0	27.4	5.62	0.4870
2023-05-24_KaskMojito3_pYR_02	pYR	133.8	27.1	5.87	0.4798
2023-05-24_KaskMojito3_pYR_01	pYR	128.0	27.0	6.06	0.4736
Mean	pYR	130.3	27.2	5.85	0.4801
Coefficient of Variance	pYR	2%	1%	4%	1%
2023-05-24_KaskMojito3_pZR_06	pZR	135.8	29.7	9.67	0.6795
2023-05-24_KaskMojito3_pZR_05	pZR	133.4	28.4	8.15	0.6549
2023-05-24_KaskMojito3_pZR_04	pZR	183.2	26.8	11.38	0.6206
Mean	pZR	150.8	28.3	9.73	0.6517
Coefficient of Variance	pZR	19%	5%	17%	5%
2023-05-25_BontragerSpecterWavecel_nYR_04	nYR	78.2	29.4	3.72	0.5289
2023-05-25_BontragerSpecterWavecel_nYR_05	nYR	83.9	30.3	3.00	0.5386
2023-05-25_BontragerSpecterWavecel_nYR_06	nYR	77.1	30.1	3.02	0.5321
Mean	nYR	79.7	29.9	3.25	0.5332
Coefficient of Variance	nYR	5%	1%	13%	1%
2023-05-25_BontragerSpecterWavecel_pXR_01	pXR	118.3	18.6	3.67	0.2928
2023-05-25_BontragerSpecterWavecel_pXR_02	pXR	119.3	16.6	3.39	0.2638
2023-05-25_BontragerSpecterWavecel_pXR_03	pXR	128.9	17.7	3.25	0.2812
Mean	pXR	122.1	17.7	3.44	0.2793
Coefficient of Variance	pXR	5%	6%	6%	5%
2023-05-25_BontragerSpecterWavecel_pYR_03	pYR	111.8	24.8	3.93	0.4441
2023-05-25_BontragerSpecterWavecel_pYR_02	pYR	109.3	24.1	3.85	0.4344
2023-05-25_BontragerSpecterWavecel_pYR_01	pYR	113.2	24.6	3.91	0.4488
Mean	pYR	111.4	24.5	3.90	0.4424
Coefficient of Variance	pYR	2%	1%	1%	2%
2023-05-25_BontragerSpecterWavecel_pZR_06	pZR	123.3	23.3	6.03	0.5581
2023-05-25_BontragerSpecterWavecel_pZR_05	pZR	117.9	25.5	5.43	0.5937
2023-05-25_BontragerSpecterWavecel_pZR_04	pZR	119.0	24.1	5.93	0.5695
Mean	pZR	120.1	24.3	5.80	0.5738
Coefficient of Variance	pZR	2%	5%	6%	3%
2023-05-25_SpecializedAlignMIPS_nYR_04	nYR	132.8	14.2	2.22	0.2522
2023-05-25_SpecializedAlignMIPS_nYR_05	nYR	134.8	14.4	2.12	0.2598
2023-05-25_SpecializedAlignMIPS_nYR_06	nYR	127.4	13.6	2.04	0.2431
Mean	nYR	131.7	14.1	2.13	0.2517
Coefficient of Variance	nYR	3%	3%	4%	3%
2023-05-25_SpecializedAlignMIPS_pXR_01	pXR	106.8	9.1	2.89	0.1731
2023-05-25_SpecializedAlignMIPS_pXR_02	pXR	112.2	12.4	3.32	0.2163
2023-05-25_SpecializedAlignMIPS_pXR_03	pXR	120.3	10.7	2.81	0.1850
Mean	pXR	113.1	10.7	3.00	0.1915
Coefficient of Variance	pXR	6%	15%	9%	12%
2023-05-25_SpecializedAlignMIPS_pYR_03	pYR	143.3	14.0	2.27	0.2451
2023-05-25_SpecializedAlignMIPS_pYR_02	pYR	141.7	15.2	2.58	0.2734
2023-05-25_SpecializedAlignMIPS_pYR_01	pYR	132.7	15.1	2.58	0.2718
Mean	pYR	139.2	14.8	2.48	0.2634
Coefficient of Variance	pYR	4%	4%	7%	6%
2023-05-25_SpecializedAlignMIPS_pZR_06	pZR	138.4	16.3	4.33	0.3322
2023-05-25_SpecializedAlignMIPS_pZR_05	pZR	142.4	17.1	4.75	0.3492
2023-05-25_SpecializedAlignMIPS_pZR_04	pZR	139.0	17.5	5.09	0.3460
Mean	pZR	139.9	17.0	4.72	0.3425
Coefficient of Variance	pZR	2%	3%	8%	3%
2023-05-26_ABUSGamechanger_nYR_04	nYR	122.4	28.9	5.20	0.5132
2023-05-26_ABUSGamechanger_nYR_05	nYR	127.0	28.8	5.53	0.5095
2023-05-26_ABUSGamechanger_nYR_06	nYR	119.3	29.1	5.23	0.5159
Mean	nYR	122.9	28.9	5.32	0.5129
Coefficient of Variance	nYR	3%	0%	3%	1%
2023-05-26_ABUSGamechanger_pXR_01	pXR	167.4	19.2	3.93	0.2894
2023-05-26_ABUSGamechanger_pXR_02	pXR	181.9	21.0	4.78	0.3216
2023-05-26_ABUSGamechanger_pXR_03	pXR	172.5	21.0	4.35	0.3242
Mean	pXR	174.0	20.4	4.35	0.3117
Coefficient of Variance	pXR	4%	5%	10%	6%
2023-05-26_ABUSGamechanger_pYR_03	pYR	151.9	27.9	7.48	0.4945
2023-05-26_ABUSGamechanger_pYR_02	pYR	154.2	27.7	7.53	0.4931
2023-05-26_ABUSGamechanger_pYR_01	pYR	149.5	27.7	6.91	0.4953
Mean	pYR	151.9	27.8	7.31	0.4943
Coefficient of Variance	pYR	2%	0%	5%	0%
2023-05-26_ABUSGamechanger_pZR_04	pZR	130.9	22.1	6.48	0.5063
2023-05-26_ABUSGamechanger_pZR_05	pZR	126.6	23.1	6.66	0.5171
2023-05-26_ABUSGamechanger_pZR_06	pZR	133.4	23.0	6.93	0.5444
Mean	pZR	130.3	22.7	6.69	0.5226
Coefficient of Variance	pZR	3%	2%	3%	4%
2023-08-02_SpecializedTacticMIPS_nYR_04	nYR	126.4	13.8	1.66	0.2472
2023-08-02_SpecializedTacticMIPS_nYR_05	nYR	117.7	13.7	1.83	0.2459
2023-08-02_SpecializedTacticMIPS_nYR_06	nYR	108.5	16.9	2.05	0.2998
Mean	nYR	117.5	14.8	1.85	0.2643
Coefficient of Variance	nYR	8%	12%	11%	12%
2023-08-02_SpecializedTacticMIPS_pXR_01	pXR	153.6	8.7	2.96	0.1885
2023-08-02_SpecializedTacticMIPS_pXR_02	pXR	126.3	9.0	2.97	0.1963
2023-08-02_SpecializedTacticMIPS_pXR_03	pXR	130.2	9.3	2.52	0.1861
Mean	pXR	136.7	9.0	2.82	0.1903
Coefficient of Variance	pXR	11%	3%	9%	3%
2023-08-02_SpecializedTacticMIPS_pYR_01	pYR	132.1	13.0	2.12	0.2279
2023-08-02_SpecializedTacticMIPS_pYR_02	pYR	130.4	12.2	1.45	0.2192
2023-08-02_SpecializedTacticMIPS_pYR_03	pYR	131.3	12.9	1.94	0.2272
Mean	pYR	131.3	12.7	1.84	0.2248
Coefficient of Variance	pYR	1%	3%	19%	2%
2023-08-02_SpecializedTacticMIPS_pZR_04	pZR	117.7	14.7	3.50	0.2970
2023-08-02_SpecializedTacticMIPS_pZR_05	pZR	121.2	15.3	4.76	0.3244
2023-08-02_SpecializedTacticMIPS_pZR_06	pZR	106.2	19.6	5.00	0.3976
Mean	pZR	115.0	16.5	4.42	0.3397
Coefficient of Variance	pZR	7%	16%	18%	15%

Mean linear risk across all helmets and repeats was highest in the pXR impact location (0.233), but lower across other impact locations (pYR: 0.205, pZR: 0.177, nYR: 0.175). The linear risk, calculated from PLA, across all tests in the pXR impact location was significantly higher than the tests in the other three impact locations [pXR > pYR: *U* = 5255.0, *p* = 0.0003; pXR > pZR: *U* = 6299.0, *p* < 0.0001; pXR > nYR: *U* = 6184.0, *p* < 0.0001]. Additionally, the linear risk in the pYR impact location was significantly higher than in the nYR and pZR locations [nYR < pYR: *U* = 2857.0, *p* = 0.0003; pZR < pYR: *U* = 2761.0, *p* = 0.0001].

Mean rotational risk across all helmets and repeats was highest in the pZR impact location (0.494) and lowest in the pXR impact location (0.107). Both YR tests produced similar mean rotational risks (pYR: 0.327, nYR: 0.342). The rotational risk, calculated from BrIC, across all tests in the pXR impact location was significantly lower than the tests in the other three impact locations [pXR < pYR: *U* = 520.0, *p* < 0.0001; pXR < pZR: *U* = 91.0, *p* < 0.0001; pXR < nYR: *U* = 600.0, *p* < 0.0001]. The rotational risk of the pZR tests was higher compared to other test locations [pZR > nYR: *U* = 6254.0, *p* < 0.0001; pZR > pYR: *U* = 6434.0, *p* < 0.0001; pXR < pZR: *U* = 91.0, *p* < 0.0001]. There was no statistically significant difference found across YR tests.

Mean overall risk across all helmets and repeats was highest in the pZR impact location (0.335), lowest in pXR (0.170) and similar across the nYR and nYR test locations (pYR: 0.266, nYR: 0.258). Across all tests, the overall risk was significantly lower in the pXR test configuration [pXR < pYR: *U* = 899.0, *p* < 0.0001; pXR < pZR: *U* = 563.0, *p* < 0.0001; pXR < nYR: *U* = 1082.0, *p* < 0.0001] and significantly higher in the pZR test configuration [pZR > nYR: *U* = 6215.0, *p* < 0.0001; pZR > pYR: *U* = 6079.0, *p* < 0.0001; pXR < pZR: *U* = 563.0, *p* < 0.0001]. There was no statistically significant difference found across YR tests.

## Appendix 3

### OLS Model Summaries

The ordinary least squares (OLS) model output used to explore the relationship between helmet type, impact location, mass, price and presence of MIPS and overall risk not adjusted for location exposure, P(injury@location) is shown (Fig. 13).
